# Hexokinase and Glucokinases Are Essential for Fitness and Virulence in the Pathogenic Yeast *Candida albicans*

**DOI:** 10.3389/fmicb.2019.00327

**Published:** 2019-02-25

**Authors:** Romain Laurian, Karine Dementhon, Bastien Doumèche, Alexandre Soulard, Thierry Noel, Marc Lemaire, Pascale Cotton

**Affiliations:** ^1^Génétique Moléculaire des Levures, UMR-CNRS 5240 Microbiologie Adaptation et Pathogénie, Université de Lyon – Université Lyon 1, Lyon, France; ^2^Laboratoire de Microbiologie Fondamentale et Pathogénicité, UMR-CNRS 5234, Université de Bordeaux, Bordeaux, France; ^3^Institut de Chimie et Biochimie Moléculaires et Supramoléculaires, Université de Lyon – Université Lyon 1, Lyon, France

**Keywords:** *Candida albicans*, hexokinase, glucokinase, glycolysis, glucose repression, hyphal transition, virulence

## Abstract

The pathogenic yeast *Candida albicans* is both a powerful commensal and a pathogen of humans that can infect wide range of organs and body sites. Metabolic flexibility promotes infection and commensal colonization by this opportunistic pathogen. Yeast cell survival depends upon assimilation of fermentable and non-fermentable locally available carbon sources. Physiologically relevant sugars like glucose and fructose are present at low levels in host niches. However, because glucose is the preferred substrate for energy and biosynthesis of structural components, its efficient detection and metabolism are fundamental for the metabolic adaptation of the pathogen. We explored and characterized the *C. albicans* hexose kinase system composed of one hexokinase (*Ca*Hxk2) and two glucokinases (*Ca*Glk1 and *Ca*Glk4). Using a set of mutant strains, we found that hexose phosphorylation is mostly performed by *Ca*Hxk2, which sustains growth on hexoses. Our data on hexokinase and glucokinase expression point out an absence of cross regulation mechanisms at the transcription level and different regulatory pathways. In the presence of glucose, *Ca*Hxk2 migrates in the nucleus and contributes to the glucose repression signaling pathway. In addition, *Ca*Hxk2 participates in oxidative, osmotic and cell wall stress responses, while glucokinases are overexpressed under hypoxia. Hexose phosphorylation is a key step necessary for filamentation that is affected in the hexokinase mutant. Virulence of this mutant is clearly impacted in the *Galleria mellonella* and macrophage models. Filamentation, glucose phosphorylation and stress response defects of the hexokinase mutant prevent host killing by *C. albicans*. By contributing to metabolic flexibility, stress response and morphogenesis, hexose kinase enzymes play an essential role in the virulence of *C. albicans*.

## Introduction

*C. albicans* is an opportunistic pathogen which exists in a relatively harmless state in the microbiota of healthy individuals. It is notably present on the mucosal surfaces composing the digestive tract (Odds, 1988; Calderone and Clancy, 2012). Perturbations of the normal microbiota, use of medical implants, or predisposing factors like diabetes can trigger *C. albicans* infection. *C. albicans* is the most common cause of fungal nosocomial infections associated with high mortality rates in immunocompromised patients (Jarvis and Martone, 1992; Perlroth et al., 2007; Armstrong-James et al., 2014).

*C. albicans* colonizes diverse host microenvironments such as skin, mucosa, blood, and organs, (Odds, 1988). Among the wide range of virulence traits, survival at 37°C, pH and osmolarity adaptation, secretion of lytic enzymes, alteration of the immune response, morphological changes, such as a transition between yeast and hyphae, occur during infection and promote host invasion (Noble et al., 2017). Another crucial factor is the metabolic capacity to assimilate host nutrients. The importance of metabolic flexibility to promote systemic infection and commensal colonization has been clearly emphasized during the past years (Wilson et al., 2009; Sandai et al., 2012; Brown et al., 2014; Childers et al., 2016). Genomic tools revealed that rapid transcriptional responses take place to set up a niche-specific carbon metabolism (Lorenz et al., 2004; Barelle et al., 2006; Bonhomme et al., 2011; Ene et al., 2012, 2013; Brown et al., 2014). Utilization of alternative non-fermentable sources through the glyoxylate and gluconeogenesis pathways is essential to support *C. albicans* proliferation *in vivo* (Lorenz and Fink, 2001; Miramón and Lorenz, 2017). However, physiologically relevant hexose sugars like glucose, galactose, and fructose are transiently available at low level in the gastrointestinal tract and only glucose (0.05–0.1%) is present in the bloodstream (Barelle et al., 2006; Pérez et al., 2013; Miramón and Lorenz, 2017). During survival in blood, invasion of kidneys and liver, expression of *C. albicans* infection-associated genes involved in glycolysis has been reported (Brown et al., 2007; Wilson et al., 2009; Pérez et al., 2013). Complete glycolytic activation by the two key transcriptional regulators Gal4p and Tye7p is required for full virulence in *Galleria mellonella* and mice (Askew et al., 2009). Glucose is the preferred substrate for ATP generation, metabolic precursors synthesis and maintenance of a reductive potential in eukaryotes (Flores et al., 2000; Rolland et al., 2001). Hence, accurate and efficient glucose detection and metabolization pathways constitute a fundamental basis for metabolic adaptation of the pathogen. In *C. albicans*, there are 20 predicted glucose transporters; one of them, *Ca*Hgt4, a high affinity sensor of the SRR pathway (Sugar Receptor Repressor), is essential for low glucose level induction of six of the *C. albicans* transporters. In addition, *Ca*Hgt4 is also required for filamentation and contributes to virulence in mice (Brown et al., 2006; Sabina and Brown, 2009).

Once detected, the initial step in glucose utilization is its transport and following transformation into a sugar phosphate. Most fungi contain at least two active hexose kinases: glucokinase and hexokinase. In *S. cerevisiae*, the hexokinase Hxk2 is the predominant glucose kinase in cells growing in high glucose conditions (Herrero et al., 1995). Both enzymes can support growth on glucose but hexokinases and glucokinases can also phosphorylate other hexoses like fructose and mannose (Cárdenas et al., 1998). The enzymatic equipment for hexose phosphorylation varies among different yeasts, although no physiological explanation for the differences has been found. A search in the genome of *C. albicans* revealed the presence of two hexokinases (*CaHXK1* and *CaHXK2*) and two glucokinase genes (*CaGLK1* and *CaGLK4*). The hexokinase *Ca*Hxk1 does not phosphorylate glucose but *N*-acetylglucosamine (GlcNAc), an extracellular carbon source present in the mucous membranes, triggering the transition between yeast and hyphal form (Yamada-Okabe et al., 2001; Rao et al., 2013). However, the hexokinase *Ca*Hxk2 and the glucokinases *Ca*Glk1 and *Ca*Glk4 have not been characterized so far and their respective role in *C. albicans* fitness and virulence has not been investigated yet. Moreover, nothing is known about the enzymatic functions of *Ca*Hxk2, *Ca*Glk1 and *Ca*Glk4 and their putative dual regulatory role in glucose repression. The preferential use of glucose by yeasts results from glucose-induced transcriptional repression via the *Ca*Snf1, essential AMP-kinase, which phosphorylates the transcription factor *Ca*Mig1 (Zaragoza et al., 2000; Corvey et al., 2005). Based on the *S. cerevisiae* model, *Ca*Mig1 should form a necessary complex with the hexokinase *Ca*Hxk2 to shuttle in the nucleus and generate the glucose repression signal (Moreno et al., 2005), but this particular step has not been described yet in *C. albicans*.

In this study, we evaluated the contribution of *Ca*Hxk2, *Ca*Glk1 and *Ca*Glk4 to the phosphorylation of hexoses and to the glucose repression process. Substantial insights in the functional consequences of hexokinase and/or glucokinases deficiency for *C. albicans* growth, various stress responses, morphological transition and virulence are also proposed.

## Materials and Methods

### Strains and Growth Conditions

*C. albicans* strains used in this study are listed in Supplementary Table S1. Strains were grown at 30°C or 37°C on YPG medium (1% yeast extract, 2% peptone, 2% glucose). When necessary glucose was added at various concentrations (from 0.01 to 2%) or replaced by other carbon sources like 2% glycerol or 2% lactate. To analyze the influence of the carbon source on *CaHXK2* and *CaGLK1/4* transcript level, early log phase cells grown in lactate 2% (OD = 1.8) were transferred to the different media containing 2% lactate, 0.1% or 2% glucose, 2% glycerol, 2% fructose, and 2% mannose. Cells were then grown for 1 h at 30°C. To check compensation mechanisms between *CaHXK2* and *CaGLK1/4* at the transcriptional level, cells were grown to early log phase on 2% glucose (YPG). To analyze the influence of the carbon source on *Ca*Hxk2-GFP and *Ca*Glk1-GFP expression, cells were grown to mid log phase in the different media containing various carbon sources. To analyze hexose transporter gene expression, cells were grown to early log phase in lactate 2% (OD = 1.8) and then transferred to 2% glucose (YPG). To analyze *CaHXK2* and *CaGLK1/4* transcript level during filamentation assays in liquid medium, cells were grown to OD = 1.8 in 0.5% glucose medium (YP) and then transferred for 30 and 60 min to 5% serum. For filamentation assays on solid medium, *C. albicans* cells were grown for 48 h at 37°C on Spider medium (1% Nutrient Broth, 1% mannitol, 0.2% KH_2_PO_4_, 2% agar) or 96 h on YP medium (1% yeast extract, 2% peptone) supplemented with 2.5 mM GlcNAc. Five percent calf serum was also used to induce the morphological switch at 37°C after a 2–5 days incubation period. The utilization of different carbon sources and sensitivity to different compounds (5 mM H_2_O_2_, 1.2 M KCl, 0.05% SDS, 5 mM caffeine) was monitored in liquid YPG at 30°C by spectrophotometry (Tecan Infinity 200 Pro Serie). Five ml of YPG inoculated with stationary phase cells were cultivated to an optical density at 600 (OD _600_) = 0.6. Ten μl of culture were used to inoculate 180 μl of YP medium containing different carbon sources or the required additives distributed in the wells of a plate. Controls lacking carbon source or specific compounds were performed. Plates were sealed with gas-permeable plastic film. OD_600_ was measured every 30 min during 48 h, with shaking at 380 rpm. Growth data were based on three independent experiments, each of which consisted of assays performed in triplicate. For growth under hypoxic conditions, aerated flasks were inoculated with an overnight culture of *C. albicans*, to OD_600_ of 0.2. Cells were grown until the beginning of the exponential phase OD_600_ ≈ 1.8. Hypoxic conditions were created by collecting and transferring cells suspension in hermetic and filled tubes. Different time points following the shift from normoxic to hypoxic growth conditions were considered (30, 60, 90, and 120 min). After appropriate time, cells were collected by centrifugation at 3,000 rpm for 5 min, washed twice with sterile water and rapidly frozen at -80°C. For each time point, three biological replicates were performed. For growth in 96-well plates, anoxic conditions were generated by adding 50 μl of mineral oil in each well. Growth values in the presence of stress and hypoxia correspond to the OD values reached after 24 h of growth for each strain.

### Construction of Mutant Strains

Mutants of the wild type strain SC5314 (Gillum et al., 1984) used in this study are listed in Supplementary Table S1. Mutant strains were constructed using the *SAT1* flipper selection cassette kindly provided by J. Morschhäuser (Reuss et al., 2004). *CaHXK2* homozygous mutant strain and the complemented strain *Cahxk2*Δ/Δ*c/c* were generated according to methods described by Reuss et al. (2004). The *Caglk1*Δ/Δ and *Caglk1glk4*Δ/Δ homozygous null mutant strains were constructed by one step cloning-free fusion PCR-based strategy. The *Cahxk2glk1*Δ/Δ mutant strain was constructed by deleting successively both *CaHXK2* alleles of the *Caglk1*Δ/Δ mutant using the *CaHXK2* deletion cassette (Supplementary Data S1). *Ca*Hxk2 and *Ca*Glk1 GFP epitope tagging was performed using a PCR-based strategy using pGFP-NAT1 as a template (kindly provided by S. Bates), (Milne et al., 2011). The appropriate mutants were identified by PCR analysis using a combination of primers outside the sites of cassette integration and internal primers.

### Yeast Transformation

*C. albicans* transformation was performed using the PEG Lithium technique (Walther and Wendland, 2003). After transformation, mixtures were incubated in YPG for 4 h at 30°C and then plated on YPG + nourseothricin 250 μg ml^-1^ (Werner BioAgent, Jena, Germany). Nourseothricin-sensitive cells were obtained according to Reuss et al. (2004). Transformants were grown overnight in YPG medium without selective pressure. Cells were plated on YPG containing nourseothricin (25 μg/ml). Small colonies containing nourseothricin-sensitive cells were selected after 2 days of growth at 30°C. Both alleles were disrupted or complemented in a similar manner after elimination of the *SAT1* flipper cassette. In the case of *in vivo* epitope tagging using pGFP-NAT1 (Supplementary Table S2) it was not possible to eliminate the *NAT1* marker, one allele was modified by transformation, only.

### Yeast Cell Extract and Immunoblotting

To prepare proteins extracts, cells were suspended in 500 μl of 0.1 M Tris-HCl buffer supplemented with 10% phenylmethylsulfonyl fluoride (PMSF) and then broken in the presence of 1.5 ml of glass beads using a FastPrep^®^-24 (MP Biomedicals) machine (five bursts 6.5 m/s for 30 s). Following this lysis step, cell extracts were centrifuged at 1,500 rpm for 10 min at 4°C. Proteins from the supernatant were quantified using NanoDrop 2000^®^.

Immunodetection conditions were as described by Rolland et al. (2006). The α-GFP antibody (monoclonal anti-mouse, Roche) and secondary antibody (mouse antibody, HRP conjugated, Bethyl Laboratories) mouse HRP were used at 1/5000^e^ and 1/10000^e^ final concentration, respectively.

### Determination of Hexose Kinase Activity

Either glucose, fructose, or mannose were used as substrates. The hexokinase II activity was measured spectrophotometrically through NADP^+^ reduction in a glucose-6-phosphate dehydrogenase-coupled reaction. Each reaction was performed in 1 ml spectrophotometer cuvette at room temperature. The final assay mixture consisted of 100 μl of 25 mM HEPES buffer pH 7.5, 100 μl of 10 mM MgCl_2_, 100 μl of 1 mM β-NADP, 500 μg of crude proteins extract, two units of glucose-6-phosphate dehydrogenase and (i) 100 μl of 10 mM D-glucose for glucose kinase activity, (ii) two units of phosphoglucose isomerase and 100 μl of 10 mM D-fructose for fructokinase activity, (iii) two units phosphomannose isomerase, two units of phosphoglucose isomerase, and 100 μl of 10 mM D-mannose for mannokinase activity. Reactions were started with the addition of 100 μl of 5 mM ATP. Absorbance was continuously recorded at 340 nm, for 10- or 15-min. Activities were obtained from the mean of three independent experiments and expressed as a percentage of the activity obtained with wild type crude protein extract. The apparent *K*m of crude extracts of the glucose kinases were determined with a final ATP concentration of 5 mM, a final concentration of glucose ranging from 1 μM to 100 mM. The NADPH apparition at 340 nm was measured using a Tecan Infinite M200 (Salzburg, Austria) microtiter plate reader at 30°C. A single well is composed of 10 μl glucose-6-P dehydrogenase (0.2 U/ml), 10 μl of 1 mM NADP^+^, 10 μl of 10 mM MgCl_2_, 10 μl of HEPES buffer (25 mM, pH 7.6) and 50 μg of crude extracts. The reaction was initiated by the addition of 10 μl of ATP (5 mM in potassium HEPES buffer). The activity was determined using a calibration curve of NADH in the range of 0–500 μM to consider the variability of the optical pathway. The parameters were obtained using Dynafit software and the rapid equilibrium approximation of the Michaelis–Menten equation (Kuzmic, 1996).

### GFP Detection by Microscopy

Yeast strain expressing the *Ca*Hxk2::GFP and *Ca*Glk1::GFP fusion proteins were grown to exponential phase (OD_600_ ≈ 0.8) in YP containing 0.05, 0.1, or 2% glucose or 2% lactate. Nuclei were stained by addition of DAPI to 10 μg/ml to the cultures and incubated at 28°C, 180 rpm for 60 min. Cells were washed twice with phosphate buffer saline (PBS) (10 mM Na_2_HPO_4_, 1.76 mM KH_2_PO_4_, 137 mM NaCl, and 2.7 mM KCl), collected by centrifugation and resuspended in 20 μl of PBS. GFP and DAPI localization were monitored in live cells cultures using a Zeiss Axioskop 2 Plus fluorescence microscope. Images were taken with a Zeiss AxioCam MR camera using AxioVision software and processed using LiveQuartz Images Editor.

### RNA Extraction and RT-q-PCR Analysis

Total RNA was extracted from cells grown to OD_600_ ≈ 1.5, by the acid phenol method (Collart and Oliviero, 2001). For reverse transcription-quantitative PCR (RT-qPCR) experiments, 10 μg of total RNA extract were treated with DNase I (Ambion). Then, ReVertAid H Minus reverse transcriptase (Thermo Scientific), was used as described by the manufacturer, to generate cDNAs. RT-qPCR experiments were performed with the CFX 96 Bio-Rad light cycler using SsoAdvanced Universal SYBR Green Supermix (Bio-Rad). Relative quantification was based on the 2Δ*CT* method using *CaACT1* (actin) as calibrator. The amplification reaction conditions were as follows: 95°C for 1 min, 40 cycles of 95°C for 15 s, 60°C for 30 s, and the final step 95°C for 10 s. A melting curve was generated at 95° for 10 s, 65°C for 5 s with an increment of 0.5°C until 95°C at the end of each PCR cycle, to verify that a specific product was amplified. Primers used in this study are presented in Supplementary Table S4.

### Infection of *G. mellonella* Larvae

For *G. mellonella* infection, overnight cultures of WT (SC5314), mutant or complemented strains of *C. albicans* were grown to stationary phase (OD_600_ = 5) in 2% YPG medium. Cells were centrifuged and washed three times with 0.9% NaCl. Larvae were infected with 10 μl of suspension (2.5 × 10^5^ cells) injected using a Hamilton syringe, between the third pair of prothoracic legs. Three replicates, each consisting of 10 insects, were carried out with survival rates measured daily for a period of 8 days. Infected larvae were incubated at 37°C in the dark. A control group injected with 10 μl of 0.9% NaCl was included. Death was determined based on the lack of motility and melanisation. Survival curves were plotted and their statistical significance were determined using the GraphPad Prism 7.0 program. *P-*values were estimated using Log rank tests. Surviving larvae were killed at -20°C after experiments following ice-anesthesia.

### Infection of Phagocytes With Yeasts

Macrophages from the J774A.1 (ATCC TIB-67) murine cell line were infected as previously described (Dementhon et al., 2012) in cRPMI medium (RPMI-1640 without phenol red and supplemented with 10% heat-inactivated fetal bovin serum, 1 mM sodium pyruvate and 2 g/l sodium bicarbonate) at 37°C under 5% CO_2_. Briefly, 2 × 10^5^ macrophages per well were adhered overnight in 96-well plates, and infected with 1 × 10^6^ Calcofluor White (CFW)-labeled yeast cells in stationary phase in cRPMI medium supplemented with 5 μg/ml CFW. Interaction was followed over a 24-h time course experiment. To count yeasts after phagocytosis, macrophages were infected with *C. albicans* strains as described in “Materials and Methods” section, using 10 macrophages to 1 yeast multiplicity of infection (MOI). After 4 h of interaction, the infected macrophages were collected after trypsin treatment, centrifuged for 10 min at 10,000 × *g*, and lysed in 1 ml of 0.2% ice-cold Triton X-100. Released yeast cells were resuspended in YPD and counted using Kova slides (Kova International). Triplicates were done for each experiment. The results are shown as the average of five independent experiments ±standard errors.

### Flow Cytometry Analysis

Flow cytometry assays were conducted as previously described (Dementhon et al., 2012) using a FACSCantoII (Becton Dickinson). Macrophage viability and the ratio of macrophages engaged in phagocytosis were determined after 30 min, 4 h and 24 h of infection with CFW-labeled yeasts. Quintuplets of each condition were done for each experiment. After trypsin treatment, macrophages were labeled with 0.2 μg/ml anti-mouse CD16-APC (a membrane stain) and 0.2 μM calcein-AM (Sigma) (a marker of active metabolism). The percentage of macrophage viability was calculated using the number of macrophages positive for both fluorescence (anti-CD16-APC and calcein-AM) when infected with yeasts compared to the control uninfected macrophages. Phagocytozing macrophages were quantified as the percentage of the double-stained macrophages also positive for CFW fluorescence. *T*-test was used to establish statistical significance with a significance level set at *P* < 0.05. Gating strategy was based on the specificity of each marker for a cell type. CFW stained yeast cells, calcein and anti-CD16-APC stained macrophages. The threshold for each fluorescence was determined based on positive and negative controls. Yeast without macrophages, in the presence of CFW, were used as positive control for CFW fluorescence. Macrophages alone in the presence of CFW were used as negative control. Cells in absence of CFW also served as negative controls. Yeast and hyphae were identified as positive for CFW fluorescence. Alive macrophages were first identified based on their double fluorescence for calcein (marker for an active metabolism) and anti-CD16-APC (membrane marker) (P5 population, Supplementary Figure S3). Then, this P5 population was analyzed for the CFW fluorescence: the triple fluorescent cells (Q2 quadrant, positive for calcein, anti-CD16-APC and CFW) are macrophages associated to yeast cells, called “phagocytozing macrophages.” Non-phagocytozing macrophages are the macrophages negative for CFW fluorescence (Q4 quadrant).

### Statistical Analysis

All experiments were performed at least three times independently. All statistical data were calculated with GraphPad Prism 7.0 software. For comparisons of multiple groups one-way ANOVA method was used. Significance of mean comparison is annotated as follow: ns, not significant; ^∗^*P* = 0.033; ^∗∗^*P* = 0.002; ^∗∗∗^*P* = 0.0002; ^∗∗∗∗^*P* < 0.0001.

### Ethics Statement

Our study was exempt from ethical approval. The *C. albicans* SC5314 strain used in this study is referenced in the ATCC collection as ATCCMYA-2876. Murine macrophages used for virulence tests were obtained from the J774A.1 cell line, referenced as ATCC TIB-67 in the ATCC collection. Insect larvae of *Galleria mellonella* were purchased from Sud-Est Appats^1^ (Quiege, France). There are no legal or ethical restrictions associated with use of *Galleria* larvae^2^.

## Results

### Deciphering the Hexose Kinase Activity in *C. albicans*

**FIGURE 1:**
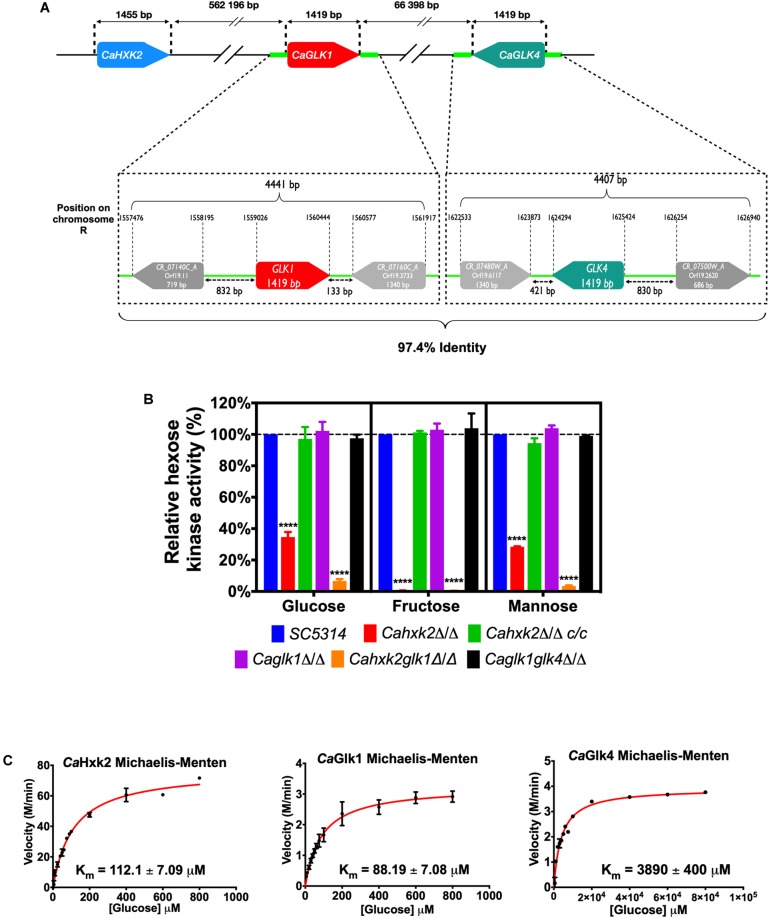
The hexose kinase system in *C. albicans.* **(A)** The hexose kinase genes are located on *C. albicans* chromosome RA (Ca22chrRA_C_albicans_SC5314:994,376…997,830). Glucokinase genes are oriented in opposite directions and bordered by highly homologous regions (light green). **(B)** Hexose phosphorylation rates in *C. albicans* wild type and hexose kinase mutant cell extracts. For each strain, the amount of glucose-6-phosphate produced was measured and expressed as a percentage of the wild type strain. Data are presented as a mean (+ standard deviation) of three independent experiments performed on three different biological samples, in triplicates (*n* = 9); ^∗∗∗∗^*P* < 0.0001; one-way ANOVA using Dunnett’s method. **(C)** Kinetic constant of hexose kinases (apparent *K*_m_ determined with crude extracts) in *C. albicans* in the presence of glucose. The experiment was performed in triplicate. Representative data are presented here.

One hypothetical hexokinase (*CaHXK2*, GenBank XM_712312) and two hypothetical glucokinase genes (*CaGlK1*, GenBank XM_705084 and *CaGLK4*, GenBank XM_707231) were found in the genomic sequence of *C. albicans*^3^. Analysis of the *C. albicans HXK2*, *GLK1* and *GLK4* sequences revealed the presence of a classical hexose kinase conserved domain organized in two regions^4^: a small and a large subdomain. The small subdomain contains the sugar-binding site of typical hexose kinases: -LGFTFSF/YP- (Bernardo et al., 2007). Protein function prediction^5^ revealed that *Ca*Hxk2, *Ca*Glk1 and *Ca*Glk4 may be involved in phosphate-containing compound metabolic processes, transferase activity and ATP-binding. The localization of these conserved domains is provided in Supplementary Table S3 (Bork et al., 1992; Kuser et al., 2000). Two nuclear localization sequences were identified in the hexokinase sequence: -PAQKRKGTFT- (8–17) and -QKRGYKTAH- (405–413). These sequences were not found in the glucokinase sequences. Both glucokinase and hexokinase genes are located on chromosome R (Figure 1A), spaced by 70 Kbp and oriented in opposite directions. The *CaGLK1* and *CaGLK4* sequences share 98.6% and 99.2% identity at the nucleotide and amino acid level, respectively. Alignment of the *CaGLK1* and *CaGLK4* genomic regions showed that a high level of identity (98%) spanned from 1,500 bp before and after the coding sequences. Within these conserved regions, separated by a few hundred of base pairs, both glucokinase genes are framed by two uncharacterized coding sequences. Alignment of these sequences, spanning the 5′ and 3′ regions of both glucokinases, revealed a level of 95.1% and 98.8% identity, respectively. This strongly indicates that the whole conserved region containing the *CaGLK1* and *CaGLK4* genes has been duplicated and conserved.

To identify the function of *CaHXK2*, *CaGLK1*, and *CaGLK4*, a set of gene disruption strains was constructed in the *C. albicans* wild type strain SC5314. Single homozygous null *Cahxk2* (*Cahxk2*Δ/Δ) and *Caglk1* (*Caglk1*Δ/Δ) mutants and double homozygous null *CaHXK2 CaGLK1* (*Cahxk2glk1*Δ/Δ) and *CaGLK1 CaGLK4* (*Caglk1glk4*Δ/Δ) mutants were constructed by replacing both wild type alleles using the excisable *CaSAT1* flipper cassette (Reuss et al., 2004). A *CaHXK2* complemented strain (*Cahxk2*Δ/Δ *c/c*) was also constructed by reintegrating the wild type coding sequence at the *HXK2* locus, using the same strategy (Supplementary Data S1).

To investigate the contribution of *Ca*Hxk2, *Ca*Glk1, and *Ca*Glk4 to the phosphorylation of hexoses in *C. albicans*, we measured the hexose kinase activity displayed by the wild type strain (SC5314) and the generated mutant strains (Figure 1B). Data obtained with *Cahxk2*Δ/Δ cell extracts revealed that glucose kinase and mannose kinase activities decreased by 65% and 75%, respectively, while the phosphorylation of fructose was totally abolished. This suggests that other enzymes, like glucokinases, could phosphorylate glucose and mannose, while fructose is phosphorylated by *Ca*Hxk2 only. Values obtained with the complemented strain *Cahxk2*Δ/Δ*c/c*, statistically comparable to the data from the wild type strain, indicated that the lack of fructose phosphorylation was due to the deletion of the gene. Deletion of one or both glucokinase genes (*CaGLK1*, *CaGLK4*) had no apparent consequence on the level of hexose phosphorylation, suggesting that glucokinase activity could be compensated by the hexokinase activity. Hexose kinase activity measured in the *Cahxk2*Δ/Δ strain corresponds to the sum of the activities of *Ca*Glk1 and *Ca*Glk4 (33% of the total activity). Glucose kinase activity, measured in the double mutant strain *Cahxk2glk1*Δ/Δ, which corresponds to the activity of *Ca*Glk4, was drastically reduced compared to the *Cahxk2*Δ/Δ mutant. This activity corresponds to 6% of the total glucose kinase activity. Taken together these results suggest that glucokinases enzymes contribute unevenly and seem to play a minor role in glucose phosphorylation in the wild type strain.

To further investigate the specificity of *Ca*Hxk2, *Ca*Glk1, and *Ca*Glk4, we determined their apparent Michaelis constant for glucose (Figure 1C). Apparent *K*_m_ of *Ca*Hxk2 was measured in the *Caglk1glk4*Δ/Δ strain, while the *K*_m_ of Glk4 was determined in *Cahxk2glk1*Δ/Δ strain. The apparent *K*_m_ of Glk1 was estimated in the *Cahxk2*Δ/Δ by subtracting the effect of *Ca*Glk4. Data revealed that hexokinase 2 and glucokinase 1 have much lower *K*_m_ values (*K*_m_ 104.87 ± 7.05 μM and *K*_m_ 84.86 ± 6.23 μM, respectively) than glucokinase 4 (*K*_m_ 3900 ± 400 μM), using glucose as a substrate. The low glucose affinity of *Ca*Glk4 could partially explain the poor contribution of this protein to glucose and mannose phosphorylation.

### Hxk2 Mostly Sustains Growth in the Presence of Hexoses

**FIGURE 2:**
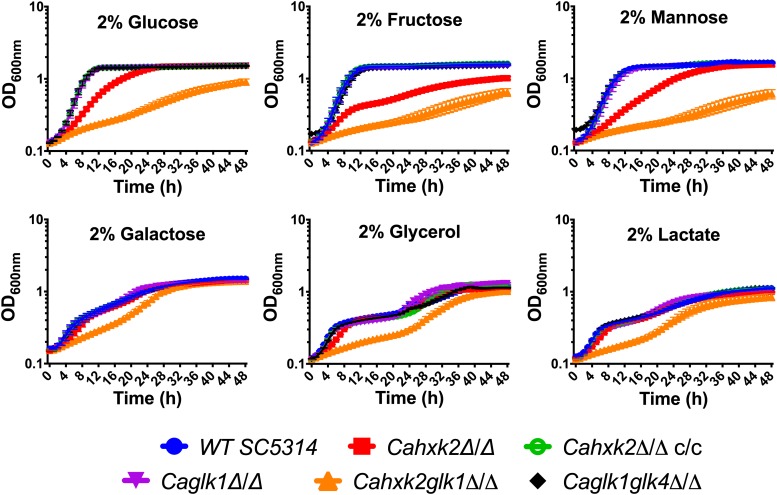
Hexokinase 2 is necessary to sustain *C. albicans* growth. Five ml of YPG inoculated with stationary phase cells were cultivated to OD_600_ = 0.6. Ten μl of culture were used to inoculate 180 μl of YP medium containing different carbon sources. A 96-well plate containing appropriate medium was inoculated with each strain at starting OD_600_ = 0.2. Cells growth was performed at 30°C and recorded during 48 h using microplates reader (TECAN infinite pro200). Data are presented as a mean (± standard deviation) of three independent experiments performed on three different biological samples, in triplicates (*n* = 9).

Impact of hexokinase and glucokinase gene deletion on growth in the presence of hexoses was evaluated (Figure 2). Delayed growth of the hexokinase mutant *Cahxk2*Δ/Δ on glucose and mannose and severely impaired growth on fructose, confirmed the absence of a functional hexokinase. Slow growth on glucose and mannose was consistent with the presence of an additional glucokinase activity. The strong growth defect observed on fructose for this mutant confirmed the fact that fructose is phosphorylated by *Ca*Hxk2 only. The residual growth observed on fructose could be due to the metabolism of the alternative carbon sources present in YPG. Growth of the mutant *Cahxk2*Δ/Δ was not affected in the presence of glycerol or galactose, substrates that are not phosphorylated by *Ca*Hxk2. This indicates that growth defects are linked to an impaired phosphorylation of hexoses. Moreover, growth of the complemented strain was comparable to the wild type. Altogether, these data clearly show that the hexokinase *Ca*Hxk2 is necessary for proper growth in *C. albicans* in the presence of glucose, fructose and mannose.

Deletion of *CaGLK1* or both *CaGLK1* and *CaGKLK4* did not affect growth. Growth of the double mutant *Cahxk2glk1*Δ/Δ was drastically affected in the presence of glucose, fructose and mannose. Growth failure was also observed, but less pronounced, in the presence of carbon sources that are not phosphorylated by hexokinase or glucokinase (galactose, lactate, glycerol). This strongly suggests that the presence of *Ca*Glk4 alone is not sufficient to sustain growth in the presence of hexoses and that the lack of both *Ca*Hxk2 and *Ca*Glk1 could affect general physiological properties, beyond hexose phosphorylation in *C. albicans.*

### Glucokinases and Hexokinase Do Not Compensate at the Transcriptional Level and Are Differentially Regulated

**FIGURE 3:**
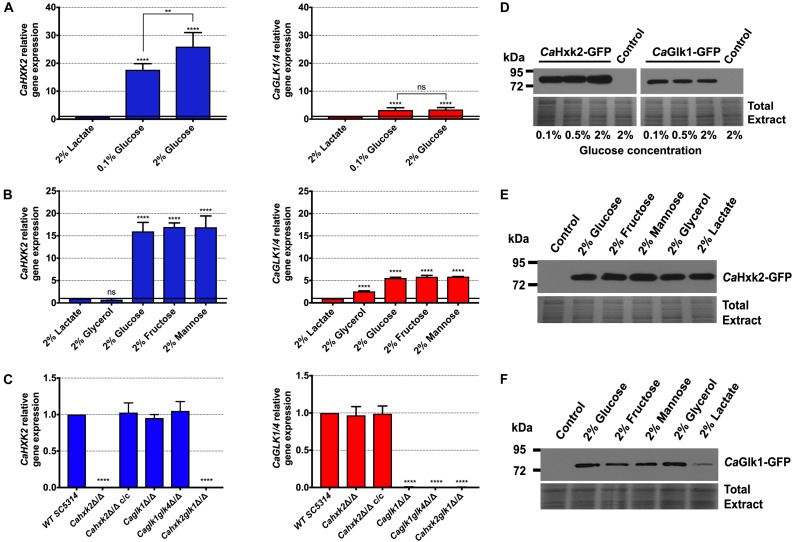
Hexokinase and glucokinases expression. **(A)** Relative expression of *CaHXK2* and *CaGLK1*/4 in wild type strain in presence of 2% lactate, 0.1% and 2% glucose. Lactate-grown cells (OD = 1.8) were transferred for 1 h to different media containing 2% carbon sources **(B)** Relative expression of *CaHXK2* and *CaGLK1*/4 in wild type strain in presence of various carbon sources (2% lactate, glycerol, glucose, fructose or mannose). For the panels **(A,B)** the results were normalized to the expression of *CaACT1*. The level of *CaHXK2* and *CaGLK1/4* mRNAs was expressed relatively to their abundance in 2% lactate, which was set to 1. Lactate-grown cells (OD = 1.8) were transferred for 1 h to different media containing 2% carbon sources **(C)** Relative expression of *CaHXK2* and *CaGLK1*/4 in wild type and hexose kinase mutant strains after growth in 2% YPG (OD = 1.8). The expression was normalized to the level of the *CaACT1* mRNA internal control. mRNA levels were expressed relatively to their abundance in the wild type strain, which was set to 1. Results represent a mean (+ standard deviation) of three independent experiments performed on three different biological samples, in duplicate (*n* = 6); ns, non-significant; ^∗∗^*P* = 0.002; ^∗∗∗∗^*P* < 0.0001. *P*-values were calculated by one-way ANOVA using Tukey’s method. **(D)** Strains of *C. albicans* expressing C-terminally GFP-tagged CaHxk2 or CaGlk1 were cultivated in presence of 0.1, 0.5, or 2% glucose to mid log phase. Whole cell lysates were analyzed for CaHxk2-GFP and CaGlk1-GFP by western blotting, using α-GFP antibody. Detection of total proteins by in-gel Coomassie staining was used as a loading control (total extract). Western blots were performed three times. Representative data are presented here. **(E,F)** *C. albicans* expressing C-terminally GFP-tagged CaHxk2 or CaGlk1 were cultivated in presence of various carbon sources (2%) to mid log phase. Whole cell lysates were analyzed for CaHxk2-GFP and CaGlk1-GFP by western blotting, using α-GFP antibody. Wild type strain protein extracts were loaded as control. Detection of total proteins by in-gel Coomassie staining was used as a loading control (total extract). Western blots were performed three times. Representative data are presented here.

To highlight the respective role of hexokinase and glucokinases, *CaHXK2*, *CaGLK1*, and *CaGLK4* expression was analyzed. To check the influence of the carbon source, cells were grown on 2% lactate and transferred to different media (Figure 3A,B). Due to the high level of homology of their coding sequences (98.6% identity) it was not possible to amplify *CaGLK1* transcripts alone. Therefore, the transcription level corresponded to the sum of *CaGLK1* and *CaGLK4* transcripts (indicated as *CaGLK1/4*). In the presence of glucose, *CaHXK2* was three times more expressed than *CaGLK1*/4 (Figure 3A). Transcription of hexokinase and glucokinase genes was strongly induced by glucose (0.1% and 2%). In the presence of glucose, *CaHXK2* was three to five times more expressed that *CaGLK1/4* (Figure 3A,B). Contrary to glucokinase genes, the level of *CaHXK2* transcripts was dependent on the glucose concentration. Transcription of hexose kinase genes was also strongly induced by mannose and fructose. Surprisingly, the transcription of glucokinase genes was induced by fructose and glycerol which are not substrates for glucokinases (Figure 3B).

To better elucidate hexose kinase gene regulation, we examined their transcription after growth on 2% glucose in the different mutant strains (Figure 3C). Expression data confirmed an absence of transcripts in the corresponding gene-deleted strains and revealed a complete restoration of the hexokinase transcription level after re-introduction of both wild type alleles. *CaHXK2* expression was not increased in the glucokinase mutants (*Caglk1*Δ/Δ, *Caglk1glk4*Δ/Δ). Likewise, *CaGLK1/4* gene expression was not increased in *Cahxk2*Δ/Δ. This suggests that unlike what happens in *S. cerevisiae* (Moreno and Herrero, 2002), no compensation mechanisms interfere to regulate glucokinases and hexokinase at the transcriptional level in the absence of one or the other gene. This points out different regulation pathways. However, this compensation could occur at the protein level since the double glucokinase mutant shows no hexose phosphorylation deficiency. Moreover, the fact that the level of *CaGLK1/4* transcripts was unchanged in the absence of the hexokinase (*Cahxk2*Δ/Δ) revealed that glucokinases genes are not subjected to glucose repression (Figure 3C). Interestingly, the level of *CaGLK4* expression detected in *Caglk1*Δ/Δ and *Cahxk2glk1*Δ/Δ was very low, just above the detection threshold. Considering that the glucokinase gene expression level detected in the mutant *Cahxk2*Δ/Δ is the sum of *GLK1* and *GLK4* transcripts, we can again assume that *CaGLK1* and *CaGLK4* are not equally expressed.

To investigate the expression of the enzymes, we constructed *HXK2::GFP* and *GLK1::GFP* strains (*CaHXK2-GFP* and *CaGLK1-GFP*) expressing *CaHXK2-GFP* and *CaGLK1-GFP* from their own promoters. GFP-tagged CaHxk2 and *Ca*Glk1 were detected in cell extracts by immunoblotting, after growth in the presence of glucose. *Ca*Hxk2-GFP and *Ca*Glk1-GFP were detected whatever the glucose concentration. However, *Ca*Hxk2-GFP was much more abundant than *Ca*Glk1-GFP (Figure 3D–F). This is consistent with the higher transcription level observed for *CaHXK2* but could also reflect a faster turnover for glucokinases. *Ca*Hxk2-GFP was equally detected in the presence of various carbon sources that are inducers of its transcription and substrates of the enzyme, but also in the presence of glycerol and lactate that do not induce *CaHXK2* transcription (Figure 3B). This could be explained by the long half-life of *Ca*Hxk2. In addition to the lowest abundance of *Ca*Glk1-GFP, the main difference between *Ca*Hxk2-GFP and *Ca*Glk1-GFP protein content was that *Ca*Glk1 was barely detectable in cell extracts after growth on lactate. This may again reflect different regulation processes for *Ca*Hxk2 and *Ca*Glk1/4.

### Hexokinase Mediates Glucose Repression but Not Glucokinases

**FIGURE 4:**
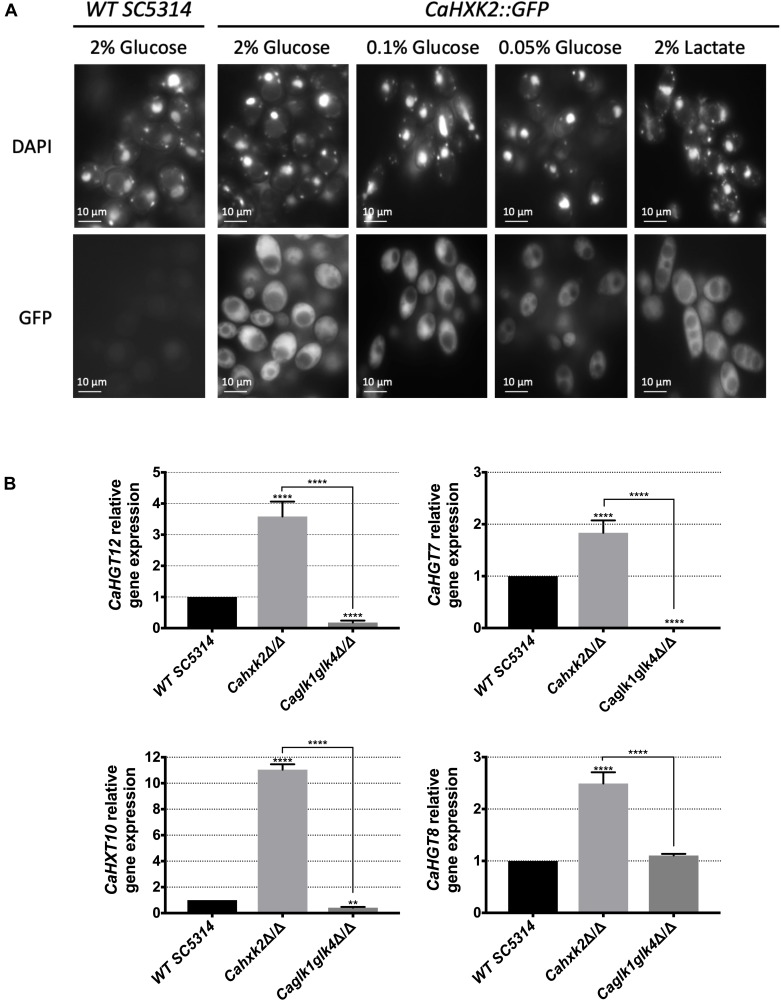
Hexokinase 2, but not glucokinases, participates to glucose repression. **(A)** Subcellular localization of CaHxk2-GFP was followed using fluorescence microscopy. Direct visualization of CaHxk2-GFP in live cells of *C. albicans* was performed as described in the “Materials and Methods” section. Nuclei were identified using DAPI staining. Transformants expressing CaHxk2-GFP were grown on medium containing 2%, 0.1%, or 0.05% glucose or 2% lactate as carbon source. GFP and DAPI localization was monitored in live cell cultures using a Zeiss Axioskop 2 Plus fluorescence microscope. Images were taken with a Zeiss AxioCam MR camera using AxioVision software. **(B)** Expression of glucose permeases, controlled by the Sugar Receptor Repressor pathway (*CaHGT12*, *CaHXT10*, and *CaHGT7*) or not (*CaHGT8*) was measured by qPCR in each strain. Cells were cultivated in the presence of 2% lactate and then transferred for 1 h in 2% glucose before RNA was extracted. Expression levels were normalized to the expression of *CaACT1*. For each permease, the mRNA levels were expressed relatively to their abundance in the wild type strain, which was set at 1. Results represent a mean (+ standard deviation) of three independent experiments performed on three different biological samples, in duplicate; (*n* = 6), ^∗∗^*P* = 0.002; ^∗∗∗∗^*P* < 0.0001. *P*-values were calculated by one-way ANOVA using Tukey’s method.

To highlight the regulatory functions of *Ca*Hxk2, we examined the localization of *Ca*Hxk2-GFP in living cells exposed to glucose (Figure 4A). Upon growth in glucose (2%) the GFP signal was distributed in all the cell (except in the vacuole) with a strong accumulation into a structure that colocalize with the nucleus. This nuclear GFP signal was less apparent in cells grown in 0.1% glucose and nearly absent in cells grown without glucose (2% lactate) or at very low glucose concentration (0.05%). This indicates that, in *C. albicans*, *Ca*Hxk2 is able to shuttle from the cytoplasm to the nucleus in presence of high glucose (0.1% and more). This observation is similar to what observed in *S. cerevisiae* grown in 2% glucose where Hxk2 is known to accumulate into the nucleus where it exerts a transcriptional regulatory function necessary for glucose repression independent of its hexokinase activity (Randez-Gil et al., 1998; Fernández-García et al., 2012). The cellular localization of the glucokinases was also investigated. Upon growth of *Ca*Glk1-GFP on 2% glucose, the GFP signal was not detected in the nucleus (Supplementary Figure S1).

To ascertain the impact of *Ca*Hxk2, *Ca*Glk1, and *Ca*Glk4 on glucose repression, we analyzed the expression of high affinity hexose transporter genes (Fan et al., 2002) that are known to be controlled by the central repressor of the glucose repression pathway, *Ca*Mig1, in response to glucose (Zaragoza et al., 2000; Sexton et al., 2007) (Figure 4B). These transporter genes (*CaHGT7*, *CaHGT12*, *CaHXT10*) are also regulated by another main glucose sensing pathway, the SRR pathway, except *CaHGT8* which is not (Brown et al., 2006). Hexose transporter gene expression was drastically enhanced (up to 10 times for *CaHXT10*) in the hexokinase mutant after transfer on 2% glucose medium. On the contrary, in the double glucokinase mutant expression level was either lowered in the case of *CaHGT7*, *CaHGT12*, and *CaHXT10* or unaffected in the case of *CaHGT8*. These data suggest that *CaHxk2* but not glucokinases, could have a repressor function on hexose transporter gene expression. Moreover, SRR-dependent *CaHGT7*, *CaHGT12*, *CaHXT10* under-expression in the glucokinase mutant may suggest an unexpected regulatory role for glucokinase in transporter gene expression.

### Hexose Kinase Enzymes Mediate Protection During Harmful Environmental Challenges: Glucokinase Contributes to the Hypoxic Response

**FIGURE 5:**
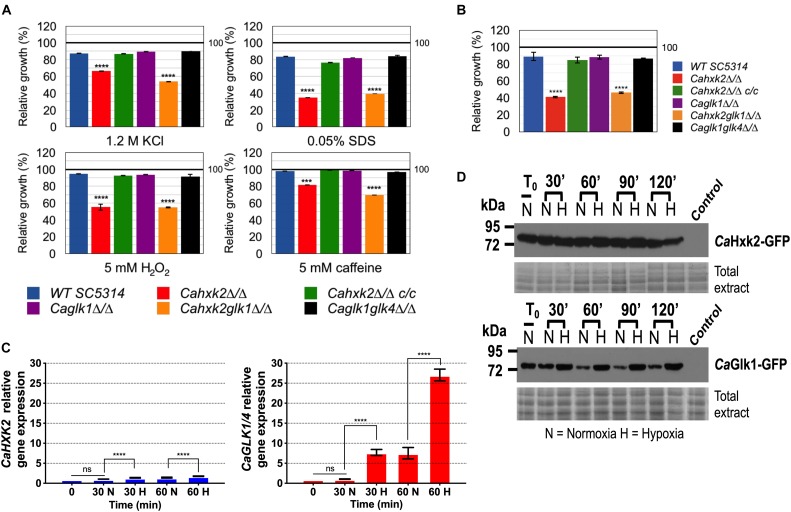
Hexose kinase enzymes mediate protection during harmful environmental challenges. **(A)** Growth of the wild type and hexose kinase mutant strains exposed to various stresses in YPG was expressed as a percentage of the growth of each strain in absence of stress which was set to 100% (black line). Data correspond to the OD values reached after 24 h of growth in microplates. **(B)** Growth of the wild type and hexose kinase mutant strains under hypoxic conditions in 2% glucose YPG was expressed as a percentage of growth in normoxia which was set to 100% (black line). **(C)** Relative expression of *CaHXK2* or *CaGLK1*/4 measured in normoxia (N) or hypoxia (H) during growth in YPG 2% glucose. Transcript level was analyzed by qPCR at 0, 30 and 60 min. Results were normalized to the *CaACT1* transcript level. The level of *CaHXK2* and *CaGLK1/4* mRNAs was expressed relatively to their abundance at time zero, which was set to 1. Histograms represents a mean of three independent experiments performed on three different biological samples, in triplicate (*n* = 9); ns, non-significant; ^∗∗∗^*P* = 0.0002; ^∗∗∗∗^*P* < 0.0001. *P*-values were calculated by one-way ANOVA using Tukey’s method. **(D)** Strains of *C. albicans* expressing CaHxk2-GFP or CaGlk1-GFP were grown in 2% glucose YPG to the mid log phase. Cells were transferred into the new medium containing 2% glucose and exposed (N = Normoxia) or not (H = Hypoxia) to oxygen. Following this shift, cells were collected at 0, 20, 60, 90, and 120 min and the detection of CaHxk2-GFP or CaGlk1-GFP was performed by western blot using α-GFP antibody. Wild type strain protein extracts were loaded as control. Detection of total proteins by in-gel Coomassie staining was used as a loading control (total extract). Western blots were performed three times. Representative data are presented here.

In *C. albicans* and number of yeasts, one strategy to counteract oxidative and osmotic stresses is the rapid endogenous synthesis of compatible solutes or, under exposure to cell wall stresses, cell wall biogenesis (Eisman et al., 2006; Sánchez-Fresneda et al., 2013). These stress responses which are directly or indirectly linked to glucose metabolism, could have been affected in the hexose kinase mutants. For that purpose, wild type and mutant strains were grown in the presence of 2% glucose (YPG) supplemented with 1.2 M KCl (osmotic stress), 5 mM H_2_O_2_ (oxidative stress), 0.05% SDS and 5 mM caffeine (cell wall stresses). Data presented Figure 5A that compare OD values of each strain in the presence and absence of stress after 24 h of growth in microplates, revealed that all stresses had an impact by decreasing growth of the hexokinase mutants (*Cahxk2*Δ/Δ, *Cahxk2glk1*Δ/Δ). Conversely, single and double glucokinase mutant strains were not significantly susceptible to the applied stresses. This suggests that *Cahxk2* is involved in stress responses through its central metabolic position.

During host infection, *C. albicans* colonizes multiple niches that greatly differ in oxygen content, meaning that it is adapted to hypoxic environments. Growth of the wild type and mutant strains under hypoxic conditions revealed the impact of *CaHXK2* deletion (Figure 5B). Growth of *Cahxk2*Δ/Δ and *Cahxk2glk1*Δ/Δ was affected by 50% after 24 h, as compared to normoxia while the deletion of one or two glucokinases had minor or no effects. The transcriptional response to hypoxia, elucidated in *C. albicans*, revealed a global upregulation of glycolytic genes (Setiadi et al., 2006; Sellam et al., 2014). This prompted us to investigate the expression of hexokinase and glucokinase in response to hypoxia (Figure 5C). After 1 h of exposure, *CaGLK1/4* transcript level increased by a factor of 25, while *CaHXK2* upregulation was much less detectable. This shows that glucokinases and hexokinase transcription is differently regulated by hypoxic conditions. This was confirmed at the protein level. GFP-tagged hexokinase was equivalently detected in normoxia and hypoxia. In contrast, immunoblot of *Ca*Glk1-GFP revealed a constant amount of protein in response to hypoxia that persisted along the growth, while in normoxia, the amount of *Ca*Glk1-GFP detected clearly decreased (Figure 5D).

### Hexose Phosphorylation by *Ca*Hxk2 Is Necessary to Filamentation

**FIGURE 6:**
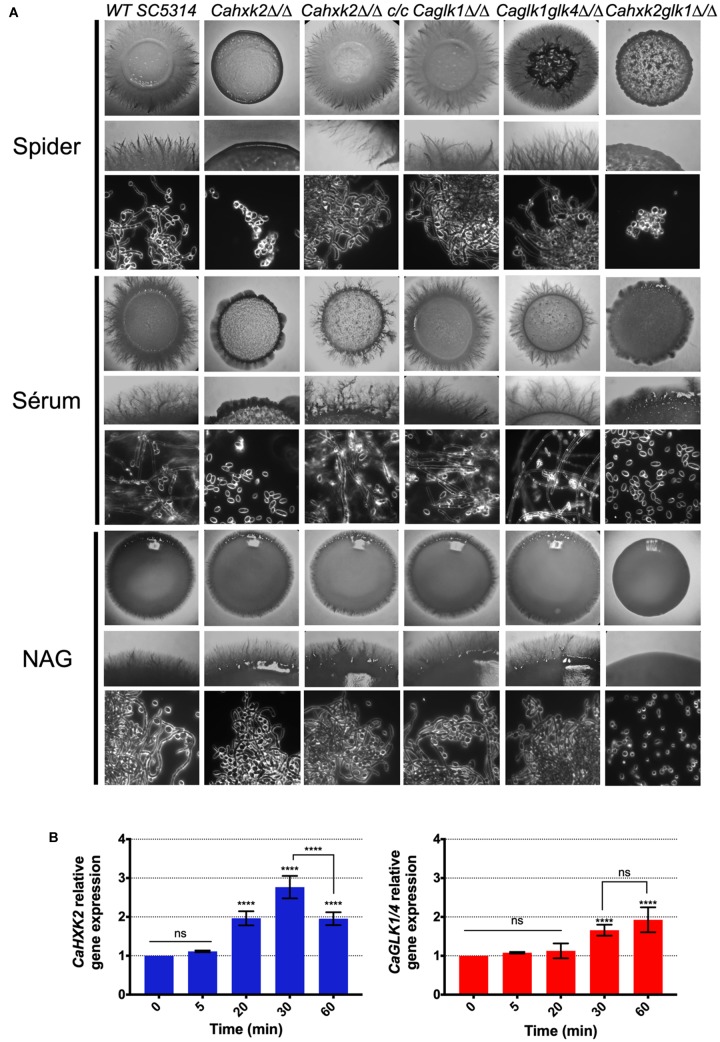
Hexose phosphorylation is required to sustain filamentation. **(A)** *C. albicans* wild type and mutant strains were grown during 3 days at 37°C on spider, serum or *N*-acetyl-glucosamine medium. For each medium, the upper and middle panels show photographs of macroscopic appearance of the colonies. Photographs present in the lower panel were obtained using Zeiss Axioskop 2 Plus microscope with dark field and show the microscopic aspect. **(B)** Relative expression of *CaHXK2* and *CaGLK1/4* in the wild type strain during filamentation after transfer from 0.5% YPG to 5% serum liquid medium. Expression level of *CaHXK2* and *CaGLK1/4* was measured by qPCR, at different time points (0, 5, 20, 30, and 60 min), and normalized to the level of the *CaACT1* mRNA internal control. mRNAs level was expressed relatively to their abundance at time zero, which was set to 1. Results represent a mean (+ standard deviation) of three independent experiments performed on three different biological samples, in duplicate (*n* = 6); ns, non-significant; ^∗∗∗∗^*P* < 0.0001. *P*-values were calculated by one-way ANOVA using Tukey’s method.

As glucose is one of the several stimuli that can trigger yeast-to-hypha development in *C. albicans* (Biswas et al., 2007), we checked the ability of hexokinase and glucokinase mutants to undergo a yeast-to-hyphae morphological transition. To evaluate the impact of the hexose phosphorylation step on filamentation, hyphal formation was induced by growth on different media containing known inducing carbon sources, requiring or not hexose kinase enzymes for further metabolization. Spider and serum media, contain, respectively, mannitol and glucose that depends upon the hexose kinase step to be metabolized. The third medium contains *N*-acetylglucosamine, that does not require *Ca*hxk2, *Ca*Glk1, or *Ca*Glk4 to be metabolized, but require *Ca*Hxk1 (Yamada-Okabe et al., 2001; Rao et al., 2013). After 2 days of growth at 37°C on serum and spider media, the wild type, *CaHXK2* complemented strain and glucokinase mutants showed abundant filaments at the periphery of the colony, while the hexokinase mutants (*Cahxk2*Δ/Δ, *Cahxk2glk1*Δ/Δ) produced hyphae-deficient colonies (Figure 6A). Morphological changes were also observed at the cell level. Microscopic observations revealed a drastically decreased proportion of filamentous structures for the hexose kinase mutant cells, suggesting that the hexokinase *CaHXK2* is necessary to the yeast-to-hyphae transition. By contrast, filamentation of the *Cahxk2*Δ/Δ hexokinase mutant was not affected during growth in the presence of *N*-acetylglucosamine. This suggests that hexose phosphorylation by *Ca*Hxk2 is an essential step for filamentation. The double mutant *Cahxk2glk1*Δ/Δ behave in a similar way to the *Cahxk2*Δ/Δ hexokinase mutant on spider and serum media but not in the presence of GlcNAc, where it appeared hypofilamentous. This filamentation defect of the double mutant *Cahxk2glk1*Δ/Δ grown on the particular carbon source GlcNAc, may be the consequence of severe physiological disturbances.

To eventually highlight a specific role in pathogenic behavior for *C. albicans* hexose kinases, we compared hexokinase and glucokinase gene expression levels during the early steps of the morphological switch. Our data did not reveal any particular transcriptional response of one gene or another (Figure 6B). Both profiles revealed a two-time increase of transcripts 30 or 60 min after the initiation of the filamentation by serum and a shift at 37°C. However, after 1 h of growth, glucokinases expression continues to increase while *CaHXK2* transcription level decreases after 30 min.

### *Cahxk2* Mutant Is Hypovirulent in *Galleria mellonella* and Macrophage Models

**FIGURE 7:**
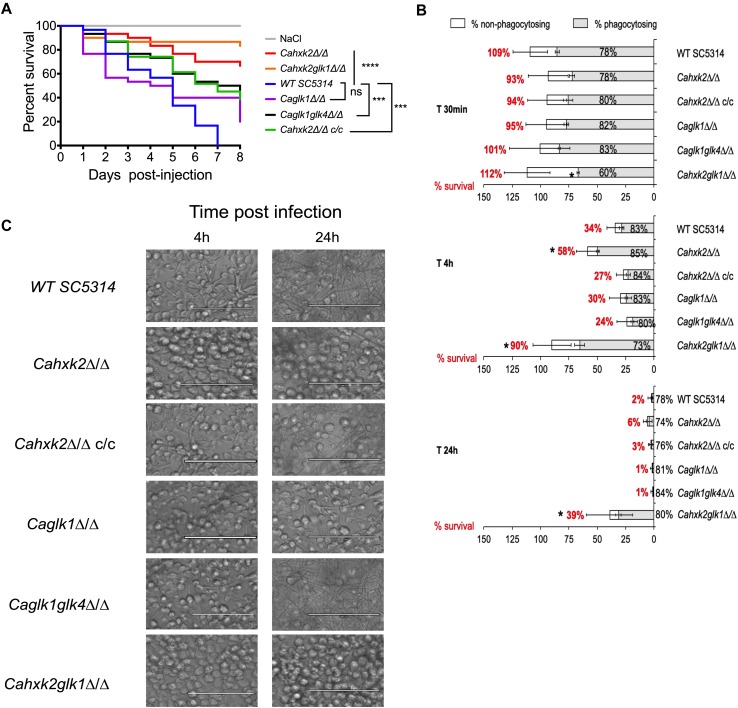
Hexokinase 2 is required for full virulence of *C. albicans*. **(A)** *Galleria mellonella* model of systemic infection. 2.5 × 10^5^ cells of wild type (*SC5314*), complemented (*Cahxk2*Δ/Δ*c/c*) or hexose kinase mutant strains were injected into the hemocoel at the last left-pro leg of 30 *Galleria* larvae. Sterile NaCl (0.9%) was injected into control larvae. Survival was monitored for 8 days at 37°C and presented in a Kaplan-Meier plot. Statistical analysis was performed using log rank tests; ns, non-significant; ^∗∗∗^*P* = 0.0002; ^∗∗∗∗^*P* < 0.0001. *Cahxk2*Δ/Δ, *Cahxk2glk1*Δ/Δ, and *Caglk1glk4*Δ/Δ mutant strains are significantly less virulent compared to the wild type strain (*P*_value_ ≤ 0.0001). The difference observed between *Caglk1*Δ/Δ mutant and the wild type strain is not significant (*P*_value_ > 0.05). The complemented strain exhibits higher virulence than hexokinase mutants but lesser than the wild type (*P*_value_ = 0.002). **(B)** Flow cytometer analysis of mouse macrophage interaction with live *C. albicans* cells in stationary phase at MOI 1:5 (1 macrophage for 5 yeasts) over a 24-h time course experiment. The horizontal bars represent the macrophage survival, indicated as a percentage on the left side of the bar. The white part of the bars represents the percentage of non-phagocytozing macrophages. The shades tones part represents the percentage of phagocytozing macrophages. Histograms represent a mean of three independent experiments performed on three different biological samples, repeated five times (+ standard deviation). **(C)** Representative pictures of the J774 macrophages after 4 and 24 h of infection with wild type and hexose kinase mutant strains in culture flasks at MOI 1:5. The scale bars represent 100 μm.

To explore the impact of altering hexokinase and glucokinases on *C. albicans* virulence, we examined first the survival rate of the host model *G. mellonella* following infection with the wild type, mutant and complemented strains (Figure 7A). *G. mellonella* survival data indicated that there was a statistically significant difference between the survival rate of larvae infected by the mutant and the wild type strains, except for the *Caglk1*Δ/Δ single mutant. Seven days post infection, 100% of the larvae were killed by the wild type strain while the survival of the larvae infected by *Cahxk2*Δ/Δ and *Cahxk2glk1*Δ/Δ was still 70% and 85%, respectively. The double glucokinase mutant was also significantly less virulent than the wild type strain, with an intermediary survival rate of 50%. The *Cahxk2*Δ/Δ*c/c* complemented strain revealed a partially restored virulence. These data suggest that strains lacking the hexokinase or both glucokinases show an affected virulence in the *G. mellonella* model.

Secondly, we analyzed the ability of the mutant to kill macrophages at different interaction times using an *in vitro* model assay (Figure 7B). Flow cytometer analysis showed that hexokinase and glucokinase gene deletions did not modify macrophages association with yeast for any strains, except for the *Cahxk2glk1*Δ/Δ double mutant which shows a slightly decreased number of recruited macrophages at the early time of infection (60% compared to approximately 80% for the other strains). This suggests that the absence of hexokinase or glucokinase has no impact on the recognition step. Survival of macrophages was severely higher when *Cahxk2*Δ/Δ and *Cahxk2glk1*Δ/Δ were tested. After 4 h in the presence of the hexokinase mutant (*Cahxk2*Δ/Δ) the number of alive macrophages was nearly twice as high as in the presence of the wild type strain. Moreover, 90% of the macrophages infected by *Cahxk2glk1*Δ/Δ were still alive after 4 h, while 34% remained alive with the wild type strain. After 24 h, 39% of the macrophages infected with *Cahxk2glk1*Δ/Δ survived, compared to only 2% with the wild type strain and 1% for the other strains. This underlines again the very affected virulence capacities of this double mutant and highlights a role for Glk1 in the absence of Hxk2. Reintegration of the wild type *CaHXK2* gene restored the killing capacities, suggesting that the virulence defect was linked to the absence of *CaHXK2*. As compared to the wild type strain, interactions performed with glucokinase mutants (*Caglk1*Δ/Δ, *Caglk1glk4*Δ/Δ) and macrophages did not reveal significant differences. Because the process of macrophage killing relies on the formation that pierce the phagocytic membrane, the morphogenesis of the strains was analyzed after 4 and 24 h of infection (Figure 7C). Our data clearly reveal that *Cahxk2*Δ/Δ and *Cahxk2glk1*Δ/Δ did not develop hyphae during macrophage infection. In order to make sure that the growth defect of the hexokinase mutant strains was not the main cause for avirulence, *C. albicans* cells were released from macrophage after 4 h of phagocytosis by cell lysis and counted (Supplementary Figure S2). As compared to the wild type, there was no significant differences in the capacity of the mutant strains to divide inside the macrophage.

Altogether, these data suggest that the virulence defect associated to the deletion of *Cahxk2* could concern the fungal escape phase rather than the recognition and initial phagocytosis step.

## Discussion

In this study, we sought to assign functions to the hexokinase and glucokinases that could potentially contribute to the fitness and virulence of *C. albicans*. We showed that hexose phosphorylation is mostly assured by *Ca*Hxk2, which mainly sustains *in vitro* growth in the presence of hexoses. Hexokinase expression is induced by glucose and higher than glucokinase expression. But proteins are both detectable even in the absence of any phosphorylable hexose. As shown for *S. cerevisiae* glycolytic enzymes, regulation is the result of a complex mixture of gene expression and metabolic effects, in order to optimize simultaneously fluxes, protein and metabolite concentrations (Daran-Lapujade et al., 2007). *C. albicans* inhabits niches containing contrasting carbon sources. Metabolic flexibility implies that alternative carbon sources and glucose are assimilated simultaneously (Barelle et al., 2006; Childers et al., 2016). The discrepancy between *C. albicans* transcriptome and proteome has been already clearly highlighted (Sandai et al., 2012). Upon glucose exposure, *Ca*Icl1 and *Ca*Pck1, enzymes involved in the assimilation of alternative carbon sources are not degraded, while their transcripts are subjected to glucose repression. We can assume that a persistent level of *Ca*Hxk2, *Ca*Glk1 and *Ca*Glk4 could promote metabolic flexibility and stress response to cope with changing microenvironments reached by the pathogen in the various host niches.

The affected growth profiles of *Cahxk2*Δ/Δ and *Cahxk2glk1*Δ/Δ indicate the limited ability of *Ca*Glk1 and *Ca*Glk4 to allow glucose utilization in the absence of *Ca*Hxk2, while normal growth was observed in the absence of *Ca*Glk1 and/*Ca*Glk4. One possible hypothesis would be a limited glucose uptake caused by the absence of hexokinase. Hence, in *S. cerevisiae* and *K. lactis* glycolytic mutants, glycolysis controls glucose signaling via the SRR pathway. The expression of several glucose-regulated genes, like hexose transporter genes, depends on a functional glycolysis, limiting therefore glucose import (Cairey-Remonnay et al., 2015). However, this control does not seem to exist in *C. albicans*. On the contrary, expression of transporter genes controlled by the SRR pathway (*HGH12, HGT7, HXT10*) was enhanced in the hexokinase mutant *Cahxk2*Δ/Δ. Therefore, the poor expression of glucokinase genes, the low intracellular concentration of *Ca*Glk1 and *Ca*Glk4 and their low participation in hexose kinase activity, could mainly explain the growth defect in the absence of hexokinase.

Moreover, glycolysis constitutes an interface between metabolism and gene transcription. For instance, glycolysis yields pyruvate which can be oxidized into acetyl-CoA, directly implicated in histone acetylation and gene expression. In stationary yeast cells, increase glucose availability leads to higher levels of acetyl-CoA synthesis, global histone acetylation, accompanied by the induction of a thousand of growth-related genes (Friis et al., 2009). The reduced glycolytic flux of the hexokinase mutant could therefore lead to transcription defects and slower growth. In all the tested conditions, the double mutant *Cahxk2glk1*Δ/Δ presented an altered phenotype. In this context, *Ca*Glk4 is the only hexose kinase enzyme present. Because of the low affinity of *Ca*Glk4 for glucose and its very low expression, growth of *Cahxk2glk1*Δ/Δ is drastically affected in the presence of hexoses. This could be explained by the lack of efficient hexose kinase enzymes. Moreover, hexokinase and glucokinase gene deletions could lead to several drastic intracellular changes. In *S. cerevisiae*, the *hxk2* mutant has a higher H^+^-ATPase activity and a lower pyruvate decarboxylase activity which coincided with an intracellular accumulation of pyruvate (Diderich et al., 2001). Absence of glucose repression could also contribute to redirect carbon flux. In *K lactis*, the identification of hexokinase-dependent proteins related to chromatin remodeling, amino acids and protein metabolism, redox maintenance and stress response reinforces the idea that glucose kinase enzymes exert broader functions than hexose phosphorylation and glucose repression (Mates et al., 2014).

Our findings reveal that the well-established bifunctional functions of Hxk2 in *S. cerevisiae* (Herrero et al., 1995; Vega et al., 2016) also exists in *C. albicans*, while glucokinases do not seem to play a role in glucose repression. We detected *Ca*Hxk2-GFP in the nuclei in 0.1% glucose-grown cells (5 mM), which corresponds to the glucose level maintained in the bloodstream and in vaginal secretions (Barelle et al., 2006; Brown et al., 2014). Glucose repression pathway via *Ca*Hxk2, could thereby promote metabolic adaptation to favor the fitness of the pathogen, even in glucose-limited host niches. In response to glucose and according to the *S. cerevisiae* model, *Ca*Hxk2 should act as a transcriptional carbon catabolite corepressor binding to *Ca*Mig1 (Ahuatzi et al., 2007). *C. albicans* has two orthologs of *Sc*Mig1, *Ca*Mig1 and *Ca*Mig2 but, to our knowledge, no functions have been assigned yet to *Ca*Mig2. Transcriptional studies realized on *C*aMig1 revealed that it regulates a unique set of genes, annotated as carbohydrate uptake and catabolism factors (Murad et al., 2001). However, works conducted on *Ca*Mig1 revealed that it has no phosphorylation sequence for the kinase *Ca*Snf1, essential for the removal of glucose repression (Petter et al., 1997; Zaragoza et al., 2000). Deletion of *Ca*Mig1 has no effect on the expression of *CaGAL1*, a glucose repressed gene (Zaragoza et al., 2000) but impacts the transcription of hexose transporter genes (Broxton et al., 2018). Moreover, CaMig1 has been recently implicated in the resistance to weak organic acids, a novel function (Cottier et al., 2015). All these evidences show that some of the molecular mechanisms involved in glucose repression in *C. albicans* remain to be elucidated, in particular concerning the direct partners of *Ca*Hxk2. Moreover, contrary to *S. cerevisiae* (Moreno and Herrero, 2002), *C. albicans* glucokinases are not subjected to glucose repression. This suggests that glucokinases are not involved into the control of glucose phosphorylation in *C. albicans* and underline their minor role in glucose metabolism.

Contrary to glucokinases, hexokinase gene deletion had an impact on various *in vitro* stress responses. Glucose has been shown to promote stress resistance and to induce some stress genes in *C. albicans* (Rodaki et al., 2009). Our data support this finding, but furthermore indicate a role for glucose phosphorylation in stress resistance. Osmotic and oxidative stresses induce storage of trehalose, glycerol, and arabitol (Sánchez-Fresneda et al., 2013). Biosynthesis of such osmolyte sugars and polyols, directly connected to the upper part of the glycolytic pathway, depends on glucose-6-phosphate availability. This was confirmed in *S. cerevisiae* where the analysis of metabolic fluxes in a Δ*hxk2* mutant revealed a synthesis of glycerol reduced by a factor of 4.5 (Raghevendran et al., 2004). Moreover, the carbon source modulates cell wall architecture and strongly influences the resistance of *C. albicans* to osmotic and cell wall stresses. Glucose and lactate-grown cells display significant differences in cell wall elasticity and ultrastructure (Ene et al., 2012). β-Glucans are major constituents of *C. albicans* cell wall. Glucan synthases assemble UDP-glucose residues produced from phosphorylated glucose (Free, 2013). Thus, any hexose phosphorylation defect would affect the cell wall and render it more sensitive to stress.

Glycolytic gene expression has been associated to the global response of *C. albicans* to hypoxia (Synnott et al., 2010). Several transcription factors are involved in hypoxia-responsive expression of glycolytic genes. Among them, the key filamentation regulator Efg1 and the transcription factors Tye7 and Gal4 contribute to the early hypoxic response (Setiadi et al., 2006; Askew et al., 2009; Bonhomme et al., 2011; Sellam et al., 2014). Contrary to *S. cerevisiae* which ferments sugars even under aerobic conditions, *C. albicans*, a crabtree-negative yeast, ferments carbohydrates under hypoxic conditions (Johnston, 1999). Our findings specify a clear and drastic effect of hypoxia on glucokinase expression at the mRNA and protein levels. Glucokinase enzymes could be part of the global early hypoxic response, as a spare wheel, to maintain a necessary glycolytic flux during fermentation conditions in oxygen-poor niches. Moreover, this data confirms the fact that hexokinase and glucokinases are not targeted by the same regulatory pathways.

Our results show that the hexokinase mutant retains the filamentation capacity when the carbon source does not require *Ca*Hxk2 to be assimilated. Thus, the filamentation-defective phenotype of the hexokinase mutant could be linked to a phosphorylation defect. The absence of one or both glucokinases has no impact on filamentation. This could be related to their low contribution to hexose phosphorylation and, concerning the spider medium, because glucokinases do not phosphorylate fructose. The activities of several major glycolytic enzymes are known to differ in yeast and hyphal forms (Schwartz and Larsh, 1982). We have shown that induction of filamentation requires upregulation of hexose kinase genes. The morphological switch to filamentous growth requires energy and carbon source, notably to build membranes and cell walls. Thereby, several links between morphogenesis and expression of metabolic genes are established in *C. albicans.* The transcription factor Efg1, part of a Ras-cAMP-PKA signaling network and involved in morphogenesis in *C. albicans*, strongly induces glycolytic and fermentation genes (Doedt et al., 2004). Moreover, mutants of the *Ca*Hgt4/*Ca*Rgt1 pathway (SRR pathway) involved in the control of gene expression in the absence of glucose display affected filamentation phenotypes (Brown et al., 2006; Sexton et al., 2007). Thus, nested pathways control simultaneously morphogenesis, glucose signaling and metabolism and by this way *Ca*Hxk2, which directly impacts on filamentation through its kinase activity.

The hypovirulence of the hexokinase mutant suggests a central function for *Ca*Hxk2. We have shown that the glucose phosphorylation step controls filamentation. Morphological switch is a determining factor of virulence in both *Galleria* and macrophage models. Hyphae are observed in the *G. mellonella* infected tissues (Fuchs et al., 2010). Histological investigations of infected larvae revealed that the SC5314 wild type strain shows a high propensity to filament, leading to gut invasion (Perdoni et al., 2014). Time-lapse microscopy studies have shown a strong correlation between intra-phagocytic hyphal growth and macrophage lysis (Bain et al., 2015). Numerous experimental data support a model by which *C. albicans* hyphae enable escape from phagocytes by growing and consequently lysing the cell (Erwig and Gow, 2016). *Ca*Hxk2 could then be required to sustain filamentation within the host cell. Moreover, the impact could be situated at the metabolic level. *C. albicans* hexokinase and double glucokinase mutants could degrade trehalose, major hemolymph sugar in *G. mellonella* larvae (Shukla et al., 2015) to recover glucose, but remain unable or less efficient to phosphorylate it, respectively. Recently, Tucey et al. (2018) revealed concomitant up-regulation of host and pathogen glycolysis, setting up glucose competition by depleting glucose. *C. albicans*-activated macrophages shift to Warburg metabolism and become dependent on glucose for survival. During macrophage infection, both *C. albicans* free cells and escaped from macrophage could trigger rapid death to the phagocytes by depleting glucose levels. The hexokinase mutant could not compete efficiently for glucose and then turn out to be hypovirulent. Affected metabolic capacities of the hexokinase mutant, along with a less effective stress resistance and adaptation to hypoxic environments could impact colonization of host environments. When hexoses are naturally available like in the gastrointestinal tract, in the bloodstream or on epithelial cell surfaces, budding growth may depend on the optimal functioning of the hexokinase. Infection and invasion of cells, tissues and organs is associated to morphological plasticity of *C. albicans*. Infection hyphae actively adhere and penetrate into epithelial cells or induce their endocytic uptake (Dalle et al., 2010; Martin et al., 2011). Thus, filamentation defects of the mutant strain *Cahxk2* could also impact on its invasion capacities. Barelle et al. (2006) showed, using GFP fusions, that glyoxylate cycle and gluconeogenic genes are repressed by physiologically relevant concentrations of glucose during kidney infection. Thus, *C. albicans* cells growing in the mouse kidney model are supposed to assimilate carbon via glycolytic metabolism. Consequently, a decreased glycolytic activity could also directly impact on the invasion and infection capacities of the pathogen.

Our data decipher the role of glucose kinase enzymes, not only as a central point of metabolism, but also as actors in regulation, stress response and morphogenesis. Altogether, those different interconnected functions influence the virulence of the yeast. Surprisingly, while the lack of glucokinases did not impact on the phenotype of the mutants, *Ca*Glk1 clearly appeared implicated in the hypoxic response. Moreover, the fact that hexose transporter gene expression level is affected in *Caglk1glk4*Δ/Δ suggests that glucokinases could be implicated in regulation processes that remain to be elucidated. Future research might provide further insights in this challenging area.

## Author Contributions

RL and PC designed and performed the experiments, analyzed the data, and wrote the manuscript. KD and BD performed the experiments, contributed to the manuscript writing and data analysis. AS, TN, and ML contributed to manuscript writing and data analysis.

## Conflict of Interest Statement

The authors declare that the research was conducted in the absence of any commercial or financial relationships that could be construed as a potential conflict of interest.

## Footnotes

1 http://www.sudestappats.fr/

2 https://www.legifrance.gouv.fr/eli/decret/2013/2/1/AGRG1231951D/jo/texte

3 http://www.candidagenome.org/

4 http://prosite.expasy.org/

5 http://bioinf.cs.ucl.ac.uk
