# Supplementary material for: Hexokinase and Glucokinases Are Essential for Fitness and Virulence in the Pathogenic Yeast Candida albicans
Source: Front Microbiol. 2019 Feb 25;10:327. doi: 10.3389/fmicb.2019.00327 (PMC6401654; doi:10.3389/fmicb.2019.00327)
Supplement: Supplementary file 1 [file Data_Sheet_1.docx]

**
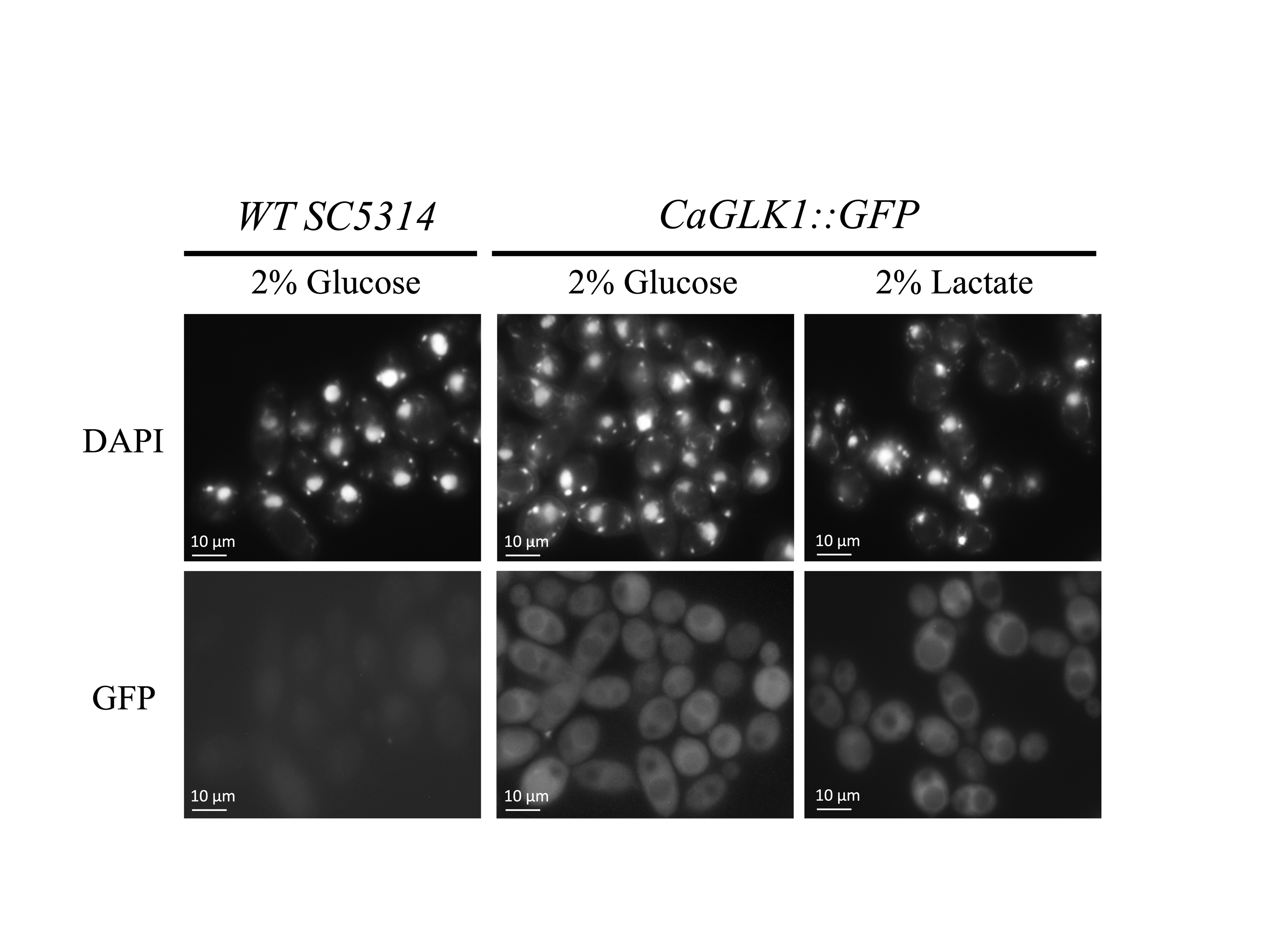
**

**Supplementary Figure S1.** **Localization of CaGlk1-GFP.** Subcellular localization of CaGlk1-GFP was followed using fluorescence microscopy. Direct visualization of CaGlk1-GFP in live cells of *C. albicans* was performed as described in the methods section. Nuclei were identified using DAPI staining. Transformants expressing CaGlk1-GFP were grown on medium containing 2% glucose or lactate as carbon source. GFP and DAPI localization was monitored in live cell cultures using a Zeiss Axioskop 2 Plus fluorescence microscope. Images were taken with a Zeiss AxioCam MR camera using AxioVision software.
