# Supplementary material for: Hexokinase and Glucokinases Are Essential for Fitness and Virulence in the Pathogenic Yeast Candida albicans
Source: Front Microbiol. 2019 Feb 25;10:327. doi: 10.3389/fmicb.2019.00327 (PMC6401654; doi:10.3389/fmicb.2019.00327)
Supplement: Supplementary file 2 [file Data_Sheet_2.docx]

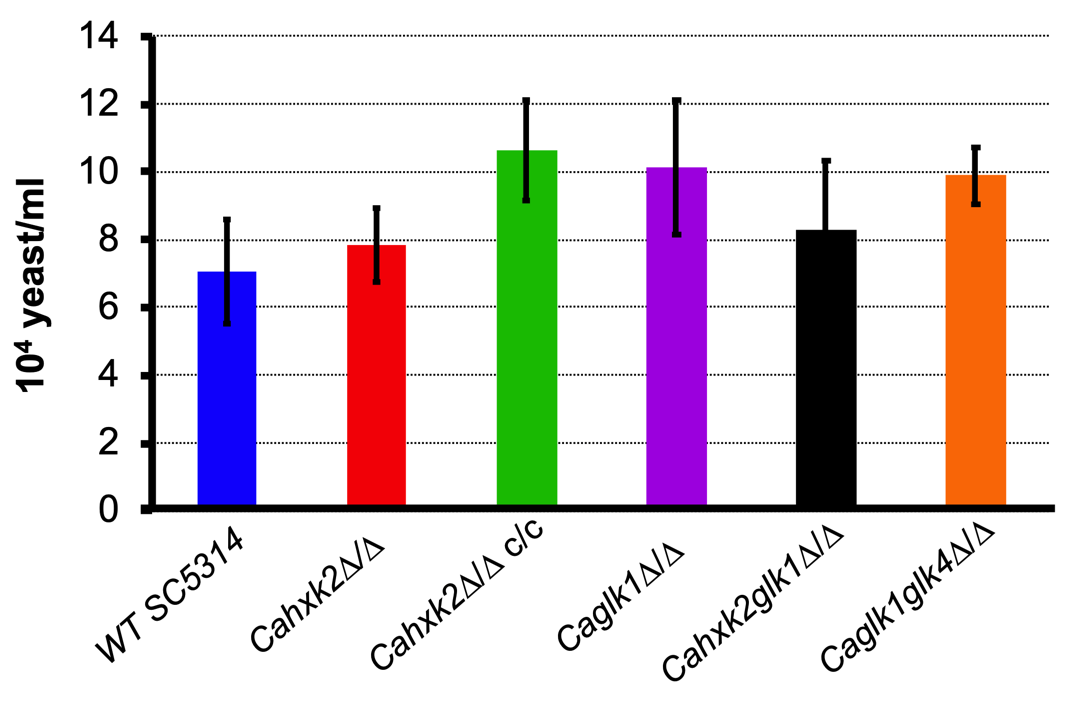


**Supplementary Figure S2.** *C. albicans* wild type and mutant strains divide equally during macrophage phagocytosis.
