# Supplementary material for: Hexokinase and Glucokinases Are Essential for Fitness and Virulence in the Pathogenic Yeast Candida albicans
Source: Front Microbiol. 2019 Feb 25;10:327. doi: 10.3389/fmicb.2019.00327 (PMC6401654; doi:10.3389/fmicb.2019.00327)
Supplement: Supplementary file 3 [file Data_Sheet_3.docx]

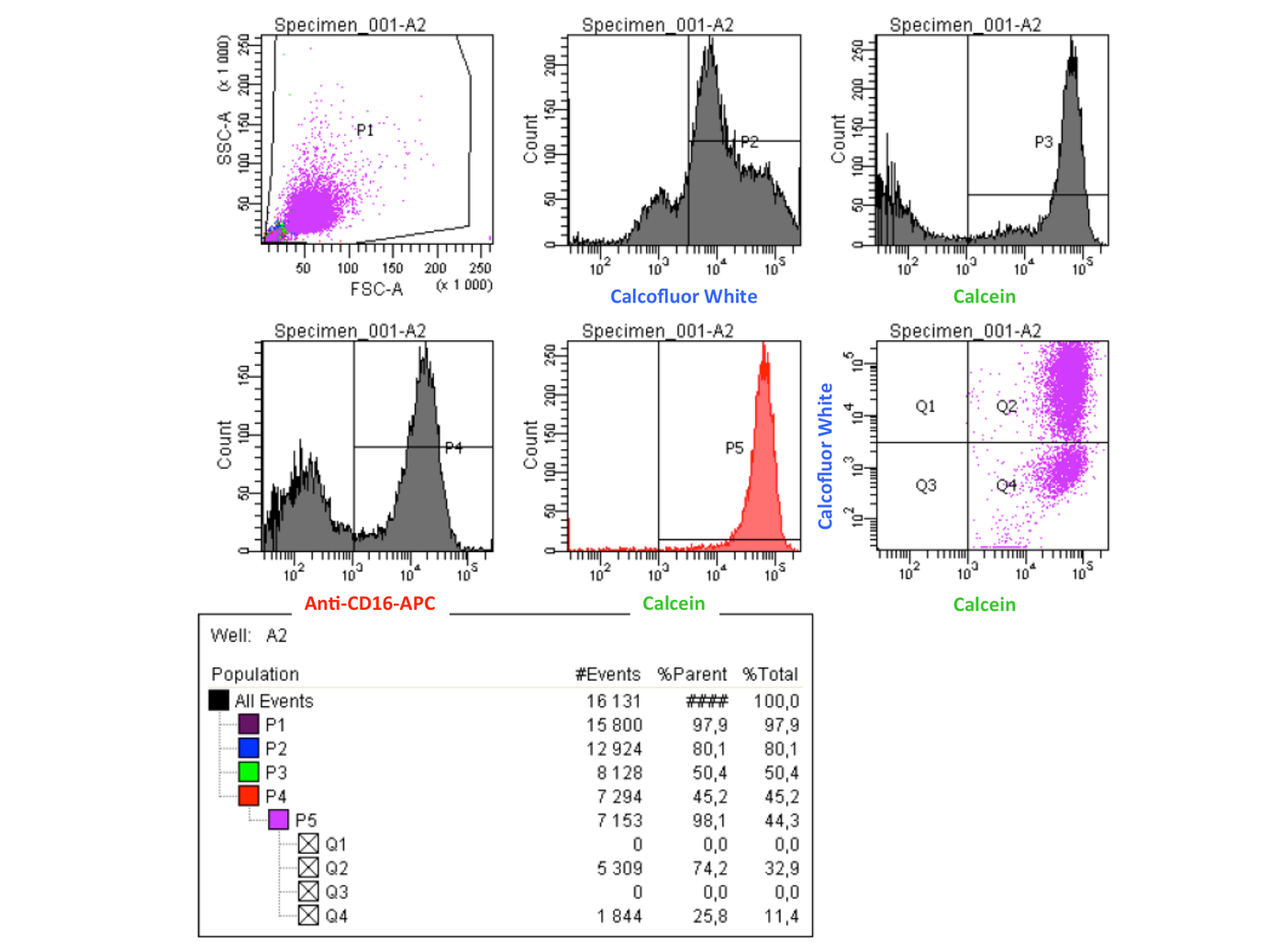


**Supplementary Figure S3 .** **Flow cytometry gating strategy.** The P5 cells, positive for both anti-CD16-APC and calcein fluorescence, are alive macrophages. Among P5 cells, those positive for Calcofluor White fluorescence (Q2 population) are engaged in phagocytosis with yeast.
