# Supplementary material for: Hexokinase and Glucokinases Are Essential for Fitness and Virulence in the Pathogenic Yeast Candida albicans
Source: Front Microbiol. 2019 Feb 25;10:327. doi: 10.3389/fmicb.2019.00327 (PMC6401654; doi:10.3389/fmicb.2019.00327)
Supplement: Supplementary file 4 [file Data_Sheet_4.docx]

**Supplementary data S1. Construction of deletion and complementation cassettes.**

For the disruption of the *CaHXK2* gene, a deletion cassette containing the *SAT1* flipper (Reuβ et al., 2004) flanked by 458 bp of the 5’ region and 484 bp of the 3’ region was assembled by short fragment homology (SFH) and cloned in pUC18 by SLIC (one step sequence and ligation independent cloning) to construct plasmid pCaPC1 (Supplementary Table S4). Primers p1 and p2 (Supplementary Table S4) were used to amplify a 458 bp upstream region (position -5 to -463). Primer p1 was flanked by 20 bp homologous to the pUC18 polylinker region, including the *SmaI* restriction site for further cloning by SLIC in pUC18. Primer p2 contained a 16 bp region complementary to the 5’ end of the *SAT1* flipper cassette. Primers p3 and p4 (Supplementary Table S4) were used to amplify the *SAT1* flipper cassette from plasmid pSFS2A (kindly provided by J. Morschhäuser). A 484 bp downstream region starting immediately after the stop codon was amplified using p5 and p6 (S4 Table). p6 was flanked by 20 pb including the *Sma*I site, situated on the opposite part of the polylinker. The upstream and downstream fragments were then assembled to the *SAT1* cassette by SFH homology using the PrimeSTAR Max DNA Polymerase (Takara) and then integrated in the *Sma*I linearized pUC18 by SLIC to generate the pCaPC1 plasmid (Supplementary Table S4). For *CaHXK2* deletion experiments pCaPC1 was digested by *Sma*I and the whole digestion mix was used for transformation experiments.

For the complementation of the *CaHXK2* gene, a *CaHXK2* genomic region spanning from -473 bp before the start codon to +363 bp after the stop codon, was amplified using primers p42 and p43 (S4 Table) containing respectively *Sph*I and *Sal*I restriction sites, to be inserted in the pUC18 vector by the SLIC technique to generate the pUCHXK2 plasmid (Supplementary Table S4). The *SAT1* flipper cassette flanked by 488 bp of the downstream *HXK2* region was amplified with primers p44 and p45 (containing respectively *Sal1* and *Xma1* restriction sites) using pCaPC1 plasmid DNA as template. This fragment was then cloned by SLIC in pUCHXK2 linearized by *Sal1* and *Xma1* to form pCaHXK2c (S3 Table)*.* For *CaHXK2* complementation experiments pCaHXK2c was digested by *Sph*1 and *Sal*1 and the whole digestion mix was used for transformation experiments.

To construct the *Caglk1∆/∆* strain*,* the *SAT1* flipper cassette was amplified from pSFS2A (Supplementary Table S2 s) using modified primers p75 and p76 (Supplementary Table S4), flanked by 80 complementary bp positioned before the start codon for the 5’ primer and before the stop codon for the 3’ primer for each gene.

For the *Caglk1glk4∆/∆* mutant*,* because of the high identity of the *CaGLK1* and *CaGLK4* nucleotide regions, both *CaGLK4* alleles were partially deleted in a *glk1∆/∆* strain, to be able to discriminate *CaGLK1* and *CaGLK4* deletions by PCR strategy. For this purpose, *CaGLK4* complementary regions of the deletion primers p100 and p101 (Supplementary Table S4), containing also complementary regions of the *SAT1* flipper cassette, were positioned 380 bp and 617 bp after the start codon, which allowed the deletion of 237 bp of the coding sequence corresponding to the hexose fixation domain.

Couples of primers p84 and p85, p88 and p89 (Supplementary Table S4) were used to amplify the *GFP* tagging cassettes, then used to target insertion to the 3’ end of *CaHXK2* and *CaGLK1*, respectively. We made sure that CaGlk1 was tagged (and not CaGlk4) by using primers p80 and p79 (Supplementary Table S4) specific from the 5’ regions of *CaGLK1* and *CaGLK4* respectively, and p82 common to both coding sequences.
