# Supplementary material for: Hexokinase and Glucokinases Are Essential for Fitness and Virulence in the Pathogenic Yeast Candida albicans
Source: Front Microbiol. 2019 Feb 25;10:327. doi: 10.3389/fmicb.2019.00327 (PMC6401654; doi:10.3389/fmicb.2019.00327)
Supplement: Supplementary file 5 [file Data_Sheet_5.docx]

| Strains | Parent | Genotype | Reference |
| --- | --- | --- | --- |
| SC5314 | - | Wild-type strain | Gillum et al., 1984 |
| *Cahxk2∆/∆* | SC5314 | *Δhxk2::FRT/ Δhxk2::FRT* | this study |
| *Cahxk2∆/∆ c/c* | *Cahxk2∆/∆* | *HXK2::FRT/ HXK2::FRT* | this study |
| *Caglk1∆/∆* | SC5314 | *Δglk1::FRT/ Δglk1::FRT* | this study |
| *Caglk1glk4∆/∆* | *Caglk1∆/∆* | *Δglk1::FRT/ Δglk1::FRT Δglk4::FRT/ Δglk4::FRT* | this study |
| *Cahxk2glk1∆/∆* | *Caglk1∆/∆* | *Δhxk2::FRT/ Δhxk2::FRT Δglk1::FRT/ Δglk1::FRT* | this study |
| *CaHXK2-GFP* | SC5314 | *HXK2/HXK2::GFP-NAT1* | this study |
| *CaGLK1-GFP* | SC5314 | *HXK2/GLK1::GFP-NAT1* | this study |

**Supplementary Table S1 .** *C. albicans* strains used in this study.
