# Supplementary material for: Hexokinase and Glucokinases Are Essential for Fitness and Virulence in the Pathogenic Yeast Candida albicans
Source: Front Microbiol. 2019 Feb 25;10:327. doi: 10.3389/fmicb.2019.00327 (PMC6401654; doi:10.3389/fmicb.2019.00327)
Supplement: Supplementary file 6 [file Data_Sheet_6.docx]

| Plasmid | Characteristics | Source or reference |
| --- | --- | --- |
| pSFS2A | pBCSK+ carrying the *SAT1* flipper cassette | Reuβ et al., 2004 |
| pGFP-NAT1 | pCR2.1 carrying a carboxyterminus GFP protein tagging cassette and the *NAT 1* resistance marker | Milne et al., 2011 |
| pCaPC1 | pUC18 carrying the *CaHXK2* deletion cassette | This study |
| pUCHXK2 | pUC18 carrying the wild type genomic region of *CaHXK2* | This study |
| pCaHXK2c | pCaPC1 carrying the wild type genomic region of *CaHXK2* | This study |

**Supplementary Table S2.** Plasmids used in this study.
