# Supplementary material for: Hexokinase and Glucokinases Are Essential for Fitness and Virulence in the Pathogenic Yeast Candida albicans
Source: Front Microbiol. 2019 Feb 25;10:327. doi: 10.3389/fmicb.2019.00327 (PMC6401654; doi:10.3389/fmicb.2019.00327)
Supplement: Supplementary file 7 [file Data_Sheet_7.docx]

**Supplementary Table S3.** Glucose and ATP binding sites and structural domains of yeast hexokinases and glucokinases

| **Kinase** | **ScHxk2** | **CaHxk2** | **ScGlk1** | **CaGlk1** |
| --- | --- | --- | --- | --- |
| Small Domain | 75-209 | 75-208 | 75-216 | 62-200 |
| Large Domain | 210-458 | 209-455 | 217-487 | 201-459 |
| Glucose binding site | Ser158, Thr175, Lys176, Asp211*, Asn237, Glu269, Glu302 | Ser157, Thr174, Lys175, Asp210*, Asn236, Glu268, Glu300 | Ser164, Thr181, Lys182, Asp218*, Asn255, Glu289, Glu323 | Ser149, Thr166, Lys167, Asp202*, Asn234, Glu260, Glu293 |
| ATP binding site   - Phosphate 1 - Connect 1 - Phosphate 2 - Adenosine - Connect 2 | 82-103 (FLAIDLGGTNLRVVLVKLGGDR)  203-223 (IEVVALINDTTGTLVASYYTD)  229-248 (GVIFGTGVNGAYYDVCSDIE)  411-439 (TGHIAADGSVYNRYPGFKEKAANALKDIY)  453-473 (IVPAEDGSGAGAAVIAALAQK) | 82-103  (YLAIDLGGTNLRVVLVKLGGNR)  202-222  (IDVVALINDTTGTLVASMYTD)  228-247  (GLIFGTGVNGAYFDVVKDIP )  411-439  (TAHCAADGSVYNKYPGFKERTAQALR)  451-471  (IVPAEDGSGVGAAVIAALTEK) | 81-102  (LLAADLGGTNFRICSVNLH)  211-230  (KVVALTNDTVGTYLSHCYTS)  247-266  (GCIFGTGTNGCYMEEINKIT)  442-471  (GEVEIGCDGSVVEYYPGFRSMLRHALALSP)  482-500  (LKIAKDGSGVGAALCALVA) | 69-90 (YLAADLGGTNFRVCSIDLKGDH)  194-214 (VKVVAIANDTVGTLLTAAYSN)  226-243  (GCIFGTGTNGAYFESKIP)  415-443 (DFEVGCDGSVIEFYPGFRQAVLESIEKIN)  453-471 (LKIAKDGSGVGAALCASTA) |
| Phosphorylation | Ser15 | Thr15^1^ | n.d.^2^ | n.d.^2^ |
| Uniprot | P04807 | P83776 | P17709 | Q59TZ8 |
| PDB | 1ig8 | n.a. ^2^ | n.a. ^2^ | n.a. ^2^ |

* catalytic residue ; ^1^: proposed residue located at the same position than Ser15 in ScHxk2 ; ^2^: not determined
