# Supplementary material for: Hexokinase and Glucokinases Are Essential for Fitness and Virulence in the Pathogenic Yeast Candida albicans
Source: Front Microbiol. 2019 Feb 25;10:327. doi: 10.3389/fmicb.2019.00327 (PMC6401654; doi:10.3389/fmicb.2019.00327)
Supplement: Supplementary file 8 [file Data_Sheet_8.docx]

**Supplementary Table S4 .** Primers used in this study.

| Name | Use | Sequence 5’->3’ |
| --- | --- | --- |
| p1 (F) | *CaHXK2* deletion | ATTCGAGCTCGGTACCCGGgctggccccatgggaataacc |
| p2 (R) | *CaHXK2* deletion | GAGGGGGGGCCCGGTAaccgagatgcaccattgtaag |
| p3 (F) | *CaHXK2* deletion | GTACCGGGCCCCCCCTCGA |
| p4 (R) | *CaHXK2* deletion | GGCCGCTCTAGAACTAGTGGATC |
| p5 (F) | *CaHXK2* deletion | GATCCACTAGTTCTAGAGCGGCCcgttggtttgttgggtgctt |
| p6 (R) | *CaHXK2* deletion | CGACTCTAGAGGATCCCCGGGcagcggtttgagaatcacttc |
| p42 (F) | *CaHXK2* complementation | CAGTGCCAAGCTTGCATGCggccccatgggaataacctg |
| p43 (R) | *CaHXK2* complementation | GGGATCCTCTAGAGTCGACgggacatcagtggtgtgtg |
| p44 (F) | *CaHXK2* complementation | CCACTGATGTCCCCGTCGACCttacaatggtgcatctcggt |
| p45 (R) | *CaHXK2* complementation | TTCGAGCTCGGTACCCGGGcagcgttgagaatcacttcatc |
| p75 (F) | *CaHXK2* complementation | AGACTGCTTTCCCAAAACTTCTTTTATTGTTTGTTCATTTAAGTTAAACTTTAAACTCTATTAATATCTATATTTCTCCATCATC gtaccgggccccccctcga |
| p76 (R) | *CaHXK2* complementation | TGCGGTACTGGCACACAATGCTGCACCAACGCCTGACCCATCTTTGGCGATCTTCAAATGAATCTTTTTATTGGTGCCCTTCAAAGggccgctctagaactagtggat |
| p79 (F) | *CaGLK1* 5’region (specific) | CAAAACTGTGTTATTGTTTCAG |
| p80 (F) | *CaGLK4* 5’region (specific) | CTATATTTCCCAGGCAAACC |
| p82 (R) | *CaGLK1/CaGLK4* coding sequence | CATAGGTTGGAATCATAGGC |
| p84 (F) | *CaHXK2* GFP tagging | GGTAGTGGTGTTGGTGCTGCCGTTATTGCTGCTTTGACCGAAAAGAGATTAAAAGAAGGTAAATCCGTTGGTTTGTTGGGTGCTggtggtggttctaaaggtgaagaattatt |
| p85 (R) | *CaHXK2* GFP tagging | ATTTTTGTCAAAACTGTCATGATACACATTACAAAACATAAAACATATCTAAACTAAAACCCAGAGTTTAAACATTGTACTTTTTcgttagtatcgaatcgacagc |
| p88 (F) | *CaGLK1* GFP tagging | CTTTGAAGGGCACCAATAAAAAGATTCATTTGAAGATCGCCAAAGATGGGTCAGGCGTTGGTGCAGCATTGTGTGCCAGTACCGCAggtggtggttctaaaggtgaagaattatt |
| p89 (R) | *CaGLK1* GFP tagging | GGTTATAATAGTCTGTATAATATTCGTATATTCAAAACTACTTTGGTCAACAATTGTAGAAATATTTGAAACCAACAcgttagtatcgaatcgacagc |
| p100 (F) | *CaGLK4* deletion | AATCCCTGTGGATTTGATGAAAGCAGAAAAGTCCAATGATTTATTTGGTTTTTTGGCAAAGAAAGTCCAATCTTTCTTACTgtaccgggccccccctcga |
| p101 (R) | *CaGLK4* deletion | TCCTGTACCAAAAATACAACCAATGATTGTGTTTCTGTTTGTCTTTGCTGAGTCATTGGAGTAAGCAGCAGTAAGCAAAGggccgctctagaactagtggat |
| pACT1 (F) | *CaACT1* amplification | ATTGTTTCCAACTGGGACGAT |
| pACT1 (R) | *CaACT1* amplification | GATGTTCTTCTGGAGCAACTCT |
| pGLK1 (F) | *CaGLK1* amplification | TTACTGCTGCTTACTCCAATGACT |
| pGFLK1 (R) | *CaGLK1* amplification | TTATCCAGCTTCTCAAAACCGTA |
| pHXK2 (F) | *CaHXK2* amplification | TTATCCAGCTTCTCAAAACCGTA |
| pHXK2 (R) | *CaHXK2* amplification | AATTGACCAGCCTTTAGTCCA |
| pICL1(F) | *CaICL1* amplification | CTACTTTATCATTGGTGCCACTAACCC |
| pICL1(R) | *CaICL1* amplification | AGACTCAATGGCAGCTAATTCGTTACC |
| pPCK1(F) | *CaPCK* amplification | GGTCCAAAAGTTGTCTCTTAATACCG |
| pPCK1(R) | *CaPCK* amplification | GCACCAGTAGATGAGATAGTAGTACC |
